# m6A Methylation Patterns and Tumor Microenvironment Infiltration Characterization in Clear-Cell Renal Cell Carcinoma

**DOI:** 10.3389/fgene.2022.864549

**Published:** 2022-04-21

**Authors:** Tianming Ma, Jiawen Wang, Xiaodong Liu, Wei Zhang, Lingfeng Meng, Yaoguang Zhang

**Affiliations:** ^1^ Department of Urology, Beijing Hospital, National Center of Gerontology, Institute of Geriatric Medicine, Chinese Academy of Medical Sciences, Beijing, China; ^2^ Graduate School of Peking Union Medical College, Chinese Academy of Medical Sciences, Beijing, China

**Keywords:** clear-cell renal cell carcinoma, N6-methyladenosine, tumor microenvironment, immune checkpoint inhibitors, pan-cancer

## Abstract

Increasing evidence suggests the essential regulation of RNA N6-methyladenosine (m6A) modification in carcinogenesis and immune response. Nevertheless, the potential impacts of these modifications on the tumor microenvironment (TME) immune cell infiltration characteristics in clear-cell renal cell carcinoma (ccRCC) remain unclear. Utilizing a consensus clustering algorithm, we determined three m6A modification patterns and identified three m6A-related gene clusters among 569 ccRCC samples, which were associated with different biological functions and clinical outcomes. Thereafter, the m6A score was constructed using m6A-associated signature genes to accurately exploit the m6A modification patterns within individual tumors. The m6A score was further demonstrated to be noticeably related to ccRCC prognosis. In addition, the m6A score was found to be strongly correlated with tumor mutational burden (TMB), microsatellite instability, immune infiltration, immune checkpoint expression, and immunotherapy response, which was also validated in the pan-cancer analyses. Our findings thoroughly elucidated that m6A modification contributes to tumor microenvironment immune-infiltrating characteristics and prognosis in ccRCC. Assessing the m6A modification patterns of individual patients with ccRCC will offer novel insights into TME infiltration and help develop more effective treatment strategies.

## Introduction

Kidney cancer is a highly prevalent malignant disease with a high mortality rate worldwide (Siegel et al., 2021; Sung et al., 2021), while clear-cell renal cell carcinoma (ccRCC) is the most common subtype, accounting for more than 70% of cases (Rini et al., 2009). Because of the difficulty in making an early diagnosis, patients with ccRCC commonly suffer slow treatment progress, recurrence, and metastasis, accompanying relevant poor survival outcomes, despite the success of immune checkpoint inhibitor (ICI) therapy (Bedke et al., 2021). Thus, investigating the specific mechanisms of cancer progression and immunotherapeutic resistance may eventually achieve survival benefits. N6-methyladenosine (m6A) is regarded as the most prominent and considerable modification of mRNAs and non-coding RNAs, which can regulate various RNA metabolism-related activities, such as degradation, decay, nuclear output, and translation (He et al., 2019). m6A regulators that modulate m6A modification, including “writers” (methyltransferases), “readers” (binding proteins), and “erasers” (demethylases), potentially participate in cancer growth, invasion and metastasis, and immunomodulatory abnormalities in multiple types of cancers, including ccRCC (He et al., 2019; Gu et al., 2021; Uddin et al., 2021). Previous studies have shown that a comprehensive recognition of the expression alteration and genetic variation of potential m6A regulators under cancer heterogeneity will be beneficial for determining favorable therapeutic targets (Zhang et al., 2020; Chong et al., 2021; Sun et al., 2021).

The tumor microenvironment (TME) is a diverse and complex system composed of cancer cells, stromal cells, and various tumor-infiltrating cells (TICs). Growing evidence indicates the crucial role of TME in tumorigenesis, progression, prognosis, and immunotherapy efficacy (Heidegger et al., 2019; Hinshaw and Shevde, 2019). Immunotherapy represented by ICIs has been suggested as a first-line treatment for advanced ccRCC, at the same time, TME is corroborated to regulate the response to ICIs (Simonaggio et al., 2021). Importantly, abundant evidence supports the close correlation between TICs and m6A modifications. For instance, depletion of METTL3 and METTL14 in colorectal cancer and melanoma reportedly increases the infiltration of CD8^+^ T cells and enhances the response to anti-PD-1 therapy (Wang et al., 2020). Han et al. elucidated that YTHDF1 promotes the translational efficiency of lysosomal cathepsins in dendritic cells, while inhibition of YTHDF1 strikingly enhances the anti-tumor response of CD8^+^ T cells and immunotherapy efficacy (Han et al., 2019). In addition, recent studies have identified the impressive role of m6A modification in reflecting TME status and predicting immunotherapy efficacy in gastric cancer (STAD), colon cancer (COAD), pancreatic cancer (PAAD), hepatocellular carcinoma (LIHC), and low-grade glioma (LGG) (Zhang et al., 2020; Chong et al., 2021; Du et al., 2021; Li et al., 2021; Sun et al., 2021). Therefore, comprehensive recognition of the infiltration characteristics of TME mediated by a variety of m6A regulators will help strengthen our comprehension of TME and immunomodulatory effects. In this study, we aimed to integrate the transcriptome RNA sequencing data of 569 ccRCC samples to comprehensively exploit m6A modification patterns and reveal the correlations between m6A modification patterns and cancer progression, prognosis, and TME characteristics.

## Methods

### Data Collection and Processing

Supplementary Figure S1 shows the flow chart of our study. Publicly available ccRCC datasets, including RNA sequencing data (in Fragments Per Kilobase per total Million mapped reads [FPKM] format), mutation data, and clinicopathological information, were collected from The Cancer Genome Atlas (TCGA) database (https://portal.gdc.cancer.gov/). Next, the FPKM format was converted into transcripts per kilobase million. After excluding patients with incomplete information, 530 patients with ccRCC were included in the analysis. A ccRCC cohort (GSE29609, *n* = 39) with detailed transcriptomic and survival data was also enrolled in the analysis, which was obtained from the Gene Expression Omnibus (GEO, https://www.ncbi.nlm.nih.gov/geo/). Finally, a total of 569 patients with ccRCC were enrolled for further analysis. Copy number variation (CNV) data were collected from the UCSC Xena data portal (http://xena.ucsc.edu/). Microsatellite instability (MSI) information was obtained from a previous study (Bonneville et al., 2017). Another ccRCC dataset, GSE22541 (including 24 samples of primary ccRCC) from GEO was used to validate the results. In addition, immunotherapy data of metastatic melanoma (GSE78220, n = 26) from GEO and comprehensive immunogenomic analyses of ccRCC from the Cancer Immunome Database (https://tcia.at/) were acquired for validation analysis. The transcriptome, mutation, and clinicopathological data of the remaining 32 cancer types in TCGA were obtained from the UCSC Xena. Detailed information on these datasets is summarized in Supplementary Table S1.

### Consensus Clustering Analysis of 23 m6A Regulators

A total of 23 m6A regulators were extracted and analyzed from our integrated datasets according to recent publications (He et al., 2019), including eight writers (*METTL3, METTL14, METTL16, WTAPI, VIRMA, ZC3H13, RBM15, RBM15B*), two erasers (*ALKBH5 and FTO*), and 13 readers (*YTHDC1, YTHDC2, YTHDF1, YTHDF2, YTHDF3, HNRNPC, FMR1, LRPPRC, HNRNPA2B1, IGFBP1, IGFBP2, IGFBP3,* and *RBMX*). We then identified the differential m6A modification patterns and classified patients with ccRCC into distinct subtypes according to the expression of 23 m6A regulators using the ConsensusClusterPlus R package, and 1,000 repetitions were established to ensure the stability of classification (Wilkerson and Hayes, 2010).

### Gene Set Variation Analysis (GSVA) and Immune Cell Infiltration Estimation

GSVA was accomplished after downloading the Hallmark and c2. cp.kegg v.7.4 gene sets to explore underlying differences in biological processes and functions among m6A modification patterns (Hänzelmann et al., 2013; Liu et al., 2022a). An adjusted *p* < 0.05 was recognized as statistically significant. Single-sample gene set enrichment analysis (ssGSEA) was performed to calculate the relative abundance of TICs in the TME of ccRCC (Charoentong et al., 2017), ssGSEA is a popular enrichment algorithm, which was extensively utilized in medical studies (Liu et al., 2022b; Liu et al., 2022c). Meanwhile, the CIBERSORT, TIMER2.0, CIBERSORT-ABS,QUANTISEQ, MCPCOUNTER, xCell, and EPIC algorithms were applied to quantify the abundance of 22 TICs to illuminate the potential calculation errors generated by diverse algorithms (Chen et al., 2018). We also used the ESTIMATE algorithm to determine the immune, stromal, and ESTIMATE scores for individuals. These scores stand for the immune and stromal components alone and the total probabilities of these components in the TME (Yoshihara et al., 2013; Liu et al., 2021a; Liu et al., 2021b).

### Differentially Expressed Gene (DEG) Identification

We used the limma R package to identify DEGs among distinct m6A clusters (Ritchie et al., 2015) and set the significance criteria to an adjusted *p*-value of <0.001. The functions of the identified DEGs were elucidated via Gene Ontology (GO) and Kyoto Encyclopedia of Genes and Genomes (KEGG) enrichment analyses using the R package clusterProfiler.

### Construction of m6A Gene Signature

To quantitatively explore the m6A modification patterns in each patient with ccRCC, the m6A gene signature was generated, which was represented as the m6A score. First, univariate Cox regression analysis was performed to determine the survival-related genes among the DEGs. We then constructed gene clusters and clustered the patients into several subgroups based on these DEGs using the consensus clustering algorithm. Thereafter, principal component analysis (PCA) was conducted to establish an m6A-associated gene signature, and principal components 1 and 2 were used for serving as signature scores. Consistent with previous studies (Zhang et al., 2020; Chong et al., 2021; Sun et al., 2021), an m6A score was defined for the individual sample using the following formula: m6A score = ∑(PC1i + PC2i), where i indicates the expression value of the *i*th m6A phenotype-related gene. Additionally, we investigated the prognostic significance and associations between the m6A score and TME characteristics and further verified the results in the GSE22541 ccRCC cohort.

### Statistical Analysis

All analyses were performed using R v.4.0.3 (http://www.R-project.org). The *t*-test or Wilcoxon rank-sum test were performed for the comparison of data between the two groups. The Kruskal–Wallis test was used to compare more than two groups. The “surv-cutpoint” function of the survminer R package was utilized to identify the optimal cut-off point, based on which the patients can be classified into high- and low-m6Ascore groups. The Kaplan-Meier method and log-rank test were used to generate survival curves for prognostic analysis and to identify statistical differences. Univariate Cox regression analysis was applied to determine the hazard ratios for m6A regulators and m6A signature-related genes. The correlations were determined using Spearman’s correlation analysis. Multivariate Cox regression analyses were applied to detect independent prognostic factors. All tests were two-sided, and statistical significance was set at *p* < 0.05.

## Results

### The Landscape of m6A Regulators in ccRCC

**FIGURE 1:**
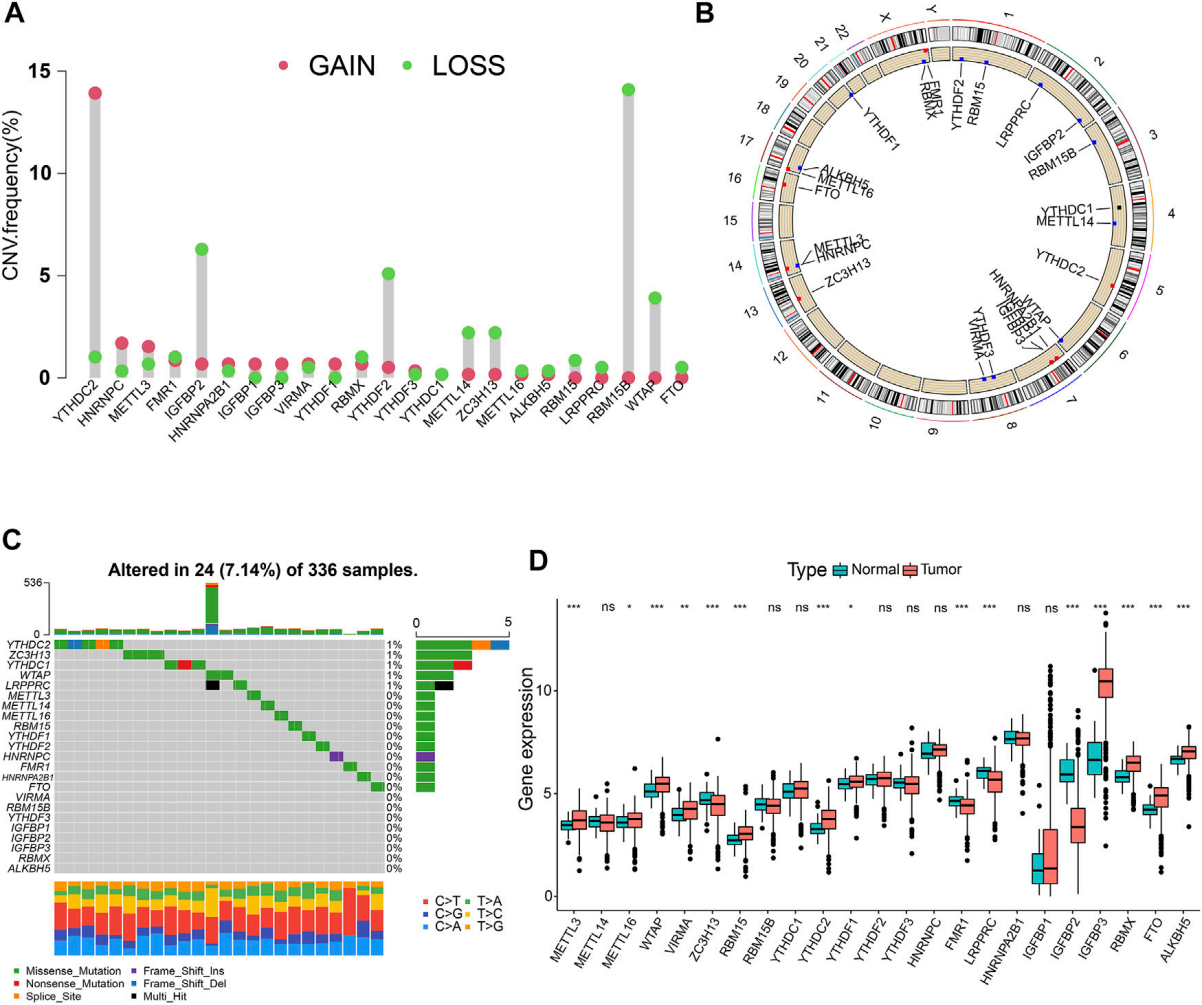
Landscape of RNA N6-methyladenosine (m6A) regulators in clear-cell renal cell carcinoma (ccRCC). **(A)** The copy number variation (CNV) frequency of 23 m6A regulators in the ccRCC cohort. **(B)** The positions of CNV changes of m6A regulators. **(C)** The mutation frequency of m6A regulators in 336 ccRCC samples. **(D)** Differential expression levels of m6A genes. *p < 0.05; **p < 0.01; ***p < 0.001; ns, not significant.

In total, 23 m6A regulators (eight writers, two erasers, and 13 readers) were investigated in this study. The CNV alteration frequency of 23 m6A regulators is shown in Figure 1A. Among them, *YTHDC2* and *RBM15B* exhibited the highest frequency of CNV in terms of amplification and deletion, respectively. Figure 1B depicts the locations of CNV mutations on 23 chromosomes for m6A regulators. We then explored the incidence of mutations and found that only 24 (7.14%) of the 336 samples experienced m6A regulator alterations in ccRCC (Figure 1C). Further analysis revealed that the expression levels of the majority of m6A regulators differed significantly between normal and ccRCC specimens (Figure 1D). Univariate Cox regression model and Spearman correlation analysis demonstrated the prognostic significance and the interactive correlations of these m6A regulators (Supplementary Figures S2A, B; Supplementary Tables S2, 3). Survival analyses using Kaplan-Meier curves also illuminated the considerable prognostic significance of these m6A regulators (Supplementary Figure S2C). These results suggest that interactions among the regulators presumably participate in the establishment of various m6A modification patterns and tumorigenesis in ccRCC.

### Identification of Three m6A Modification Patterns

**FIGURE 2:**
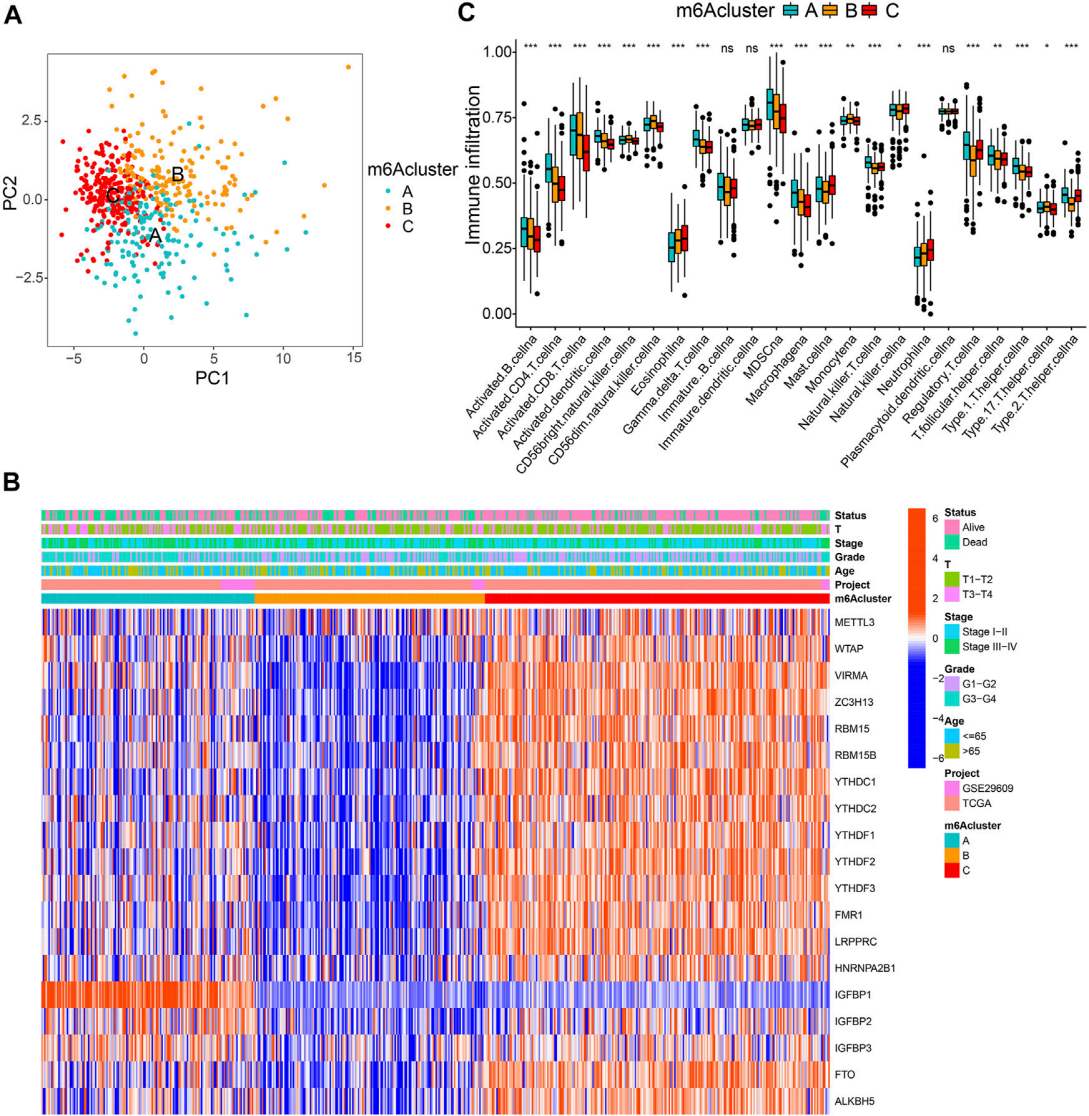
m6A clusters and relevant tumor microenvironment (TME) characteristics. **(A)** Principal component analysis of three determined m6A clusters. **(B)** Unsupervised clustering of m6A regulators. **(C)** The infiltration of TME immune cells in distinct m6A clusters.

All 569 merged samples (from two datasets: TCGA and GSE29609) were categorized into three groups according to m6A expression profiles: m6A cluster A (n = 155), m6A cluster B (n = 168), and m6A cluster C (n = 246), using a consensus clustering algorithm (Supplementary Figures S3A–C; Figure 2A; Supplementary Table S4). Among them, m6A cluster C exhibited the best prognosis (*p* < 0.001, Supplementary Figure S3D). Heatmap in Figure 2B shows the expression of m6A regulators in the three modified clusters. Using GSVA of functional genes, we summarized the biological activities of the m6A regulators. It was noticeable that some tumor hallmarks and immune activation-related processes, such as coagulation, glycolysis, mTORC1 signaling, TGF-β, Wnt-β-catenin, and inflammatory response signaling pathways were predominantly enriched in these clusters (Supplementary Figures S3E–J). Furthermore, we thoroughly evaluated the correlations among the three clusters and TICs in the ccRCC samples. There was a significant differential abundance of TICs among the three different m6A modification patterns. Among them, the proportions of several anti-tumor immune cells, such as activated CD4 T cells, activated CD8 T cells, activated dendritic cells, and T follicular helper cells were significantly higher in m6Acluster A than those in m6Acluster B/C (Figure 2C). These results indicate the potential immunomodulatory effects of m6A modification patterns.

### Generation of Three m6A-Related Gene Signatures

**FIGURE 3:**
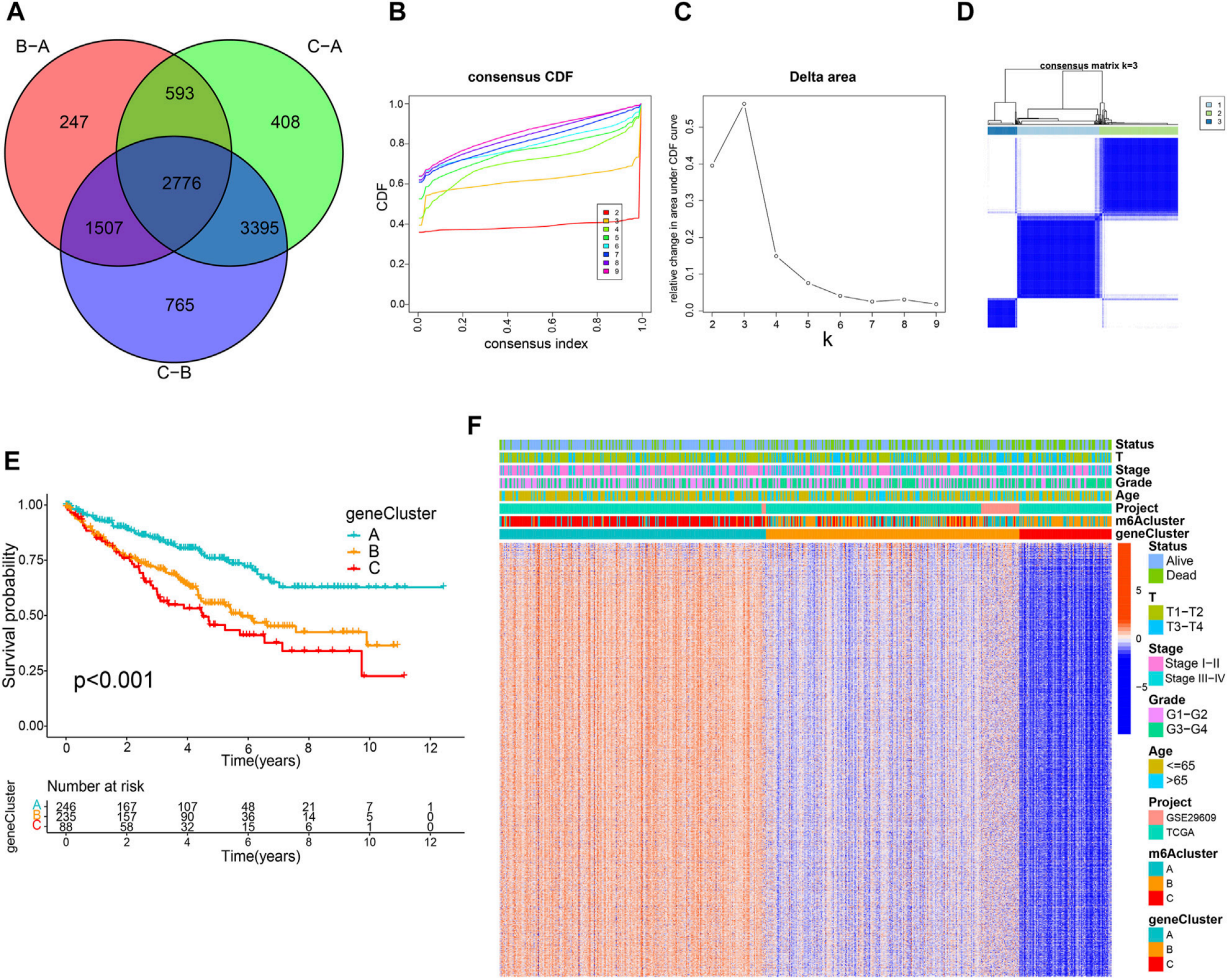
Construction of m6A-related gene signatures. **(A)** Venn diagram of 2,776 m6A-related differentially expressed genes. Consensus clustering cumulative distribution function **(B)** and delta area curves **(C)** with k = 2 to 9. **(D)** Consensus matrix. **(E)** Differential overall survival of three gene clusters. **(F)** Unsupervised clustering of m6A signature-related genes.

A total of 2,776 DEGs were identified from three m6A modification patterns using the limma R package to elucidate the potential biological characteristics of the m6A modification pattern (Figure 3A). GO and KEGG enrichment analysis demonstrated that these DEGs were associated with RNA modification, transcription, and immunity (Supplementary Figures S4A, B). Subsequently, we detected 1883 survival-related genes from 2,776 DEGs through univariate Cox regression analysis and incorporated them into the unsupervised clustering analyses; three m6A gene phenotypes were identified, named as m6A gene cluster A-C, respectively (Figures 3B–D; Supplementary Table S5). Further survival analysis suggested that cluster C exhibited the worst prognosis (*p* < 0.001) (Figure 3E). The heatmap in Figure 3F shows the transcriptome profile of these m6A clusters-related genes in three gene clusters, three m6A clusters, and clinicopathological characteristics. In addition, we compared the expression levels of m6A regulators among three gene clusters and noticed a significant difference (Supplemementary Figure S4C).

### Construction and Verification of the m6A Score

**FIGURE 4:**
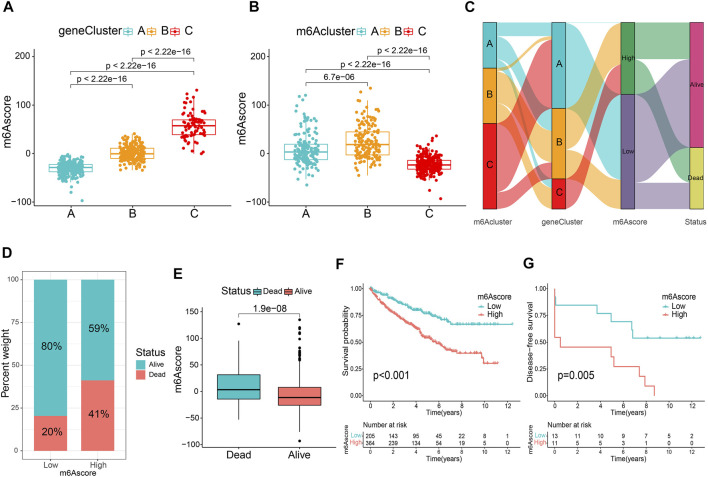
Establishment of the m6A score. Comparison of the m6A score between gene clusters **(A)** and m6A clusters **(B)**. **(C)** The alluvial diagram displaying the changes in the m6A cluster, gene cluster, m6A score and survival outcome. **(D)** The percentage weight of survival status in low or high m6A score groups. **(E)** Distribution of m6A score in dead and surviving patients. **(F)** Overall survival analysis of m6A score groups. **(G)** The application of m6A score in the GSE22541 cohort.

To accurately exploit m6A modification patterns in patients with ccRCC, the m6A score for each patient was calculated using the PCA algorithm based on the expression levels of prognostic intersected DEGs in the study. Supplementary Table S6 listed the detailed m6A score values for individual samples. Figure 4A indicates that gene cluster A had the lowest m6A score, while cluster C presented the highest m6A score. In particular, m6A cluster C showed the lowest m6A scores (Figure 4B). Then, an alluvial diagram was drawn to visualize m6A score construction (Figure 4C). Subsequently, patients were divided into low or high m6A score groups determined using the survminer package. We noticed that patient survival was significantly associated with a low m6A score (Figures 4D,E). Kaplan-Meier curves similarly demonstrated that patients in the low m6A score group had an improved overall survival (OS) than those in the high score group (*p* < 0.001, Figure 4F). Multivariate Cox regression analysis, which integrated m6A scores and several clinical characteristics, including age, sex, tumor grade, tumor stage, and T stage (NM stage was not included owing to missing data), validated that the m6A score was an independent and robust prognostic indicator for patients with ccRCC (Supplementary Figure S5A). Besides, Student’s t-test uncovered that male, G3-G4, Stage III-IV, and T3-T4 patients tended to have higher m6A scores (Supplementary Figures S5C–F), but there was no statistical difference between the two age groups (Supplementary Figure S5B). Then, stratified survival analysis based on the distinct clinicopathological factors was applied to further appraise the prediction of the m6A score. Supplementary Figures S5G–P indicate that patients with high m6A scores showed a worse OS than those with low m6A scores in every subgroup. Furthermore, the ROC analysis and the calibration curves further validated the excellent predictive value of the m6A score for predicting survival prognosis (Supplementary Figure S6). Consistent with the results above, the Kaplan-Meier curve of GSE22541 external confirmed that patients in the high m6A score group also showed poor prognoses (Figure 4G). These findings suggest that the m6A score could be used to evaluate the progression, malignancy, and survival outcomes of ccRCC.

### Characteristics of the m6A Score in Tumor Somatic Mutation

**FIGURE 5:**
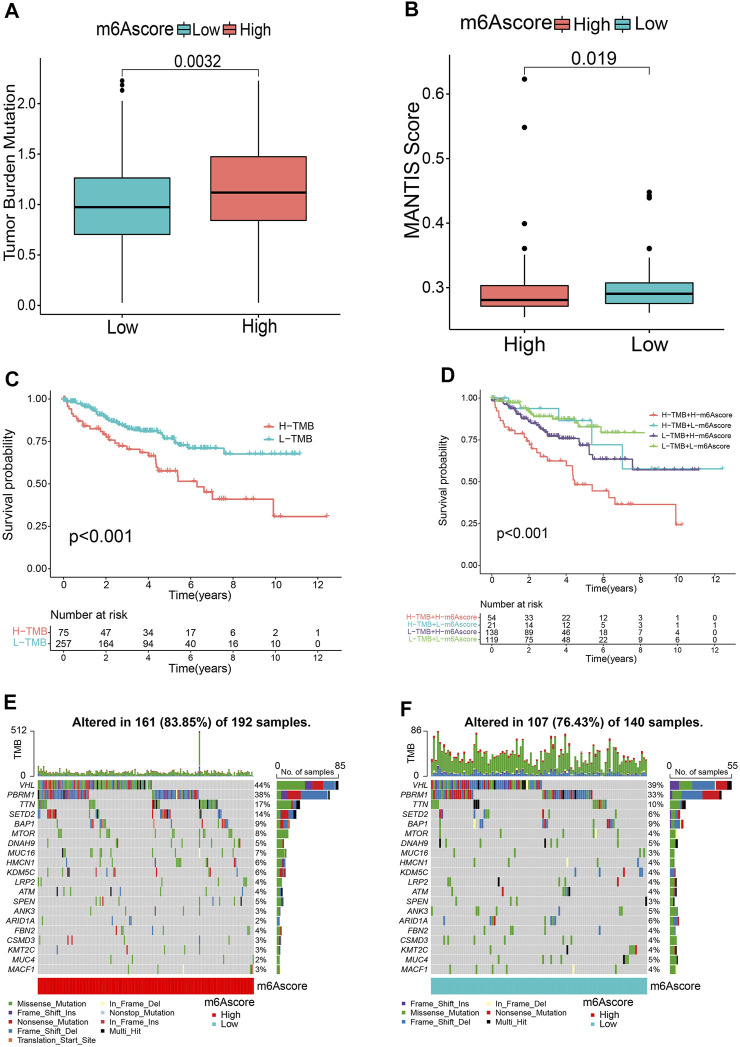
Relationship between m6A score and tumor mutational burden (TMB). Microsatellite instability (MSI) **(A)** and TMB **(B)** status between m6A score groups. **(C)** Kaplan-Meier curves revealing the survival of the low and high TMB groups. **(D)** Kaplan-Meier curves classified by both m6A score and TMB. **(E,F)** OncoPrints indicating distinct mutation conditions.

Accumulating evidence suggests that tumor mutational burden (TMB) and MSI could serve as prominent prognostic markers that are also related to immunotherapy (Bonneville et al., 2017; Samstein et al., 2019). Accordingly, we noticed that there was a significantly higher MSI in the low m6A score group (Figure 5A). We also compared the difference in TMB and observed that the high m6A score subgroup tended to present a higher TMB (Figure 5B). Next, the patients were classified into two groups based on the TMB. Kaplan-Meier curves showed that patients with low TMB scores showed a remarkable survival benefit in comparison to those with high TMB (*p* < 0.001, Figure 5C). We also found that the impact of the m6A score on OS could not be disturbed by TMB status; patients with a high m6A score always survived for a shorter period compared to those with a low m6A score (Figure 5D). Furthermore, we investigated the somatic mutation distribution differences between the two subgroups and discovered a higher mutation rate in the high m6A score group (83.85 vs. 76.43%). The results also indicated that both *VHL* (44 vs. 39%) and *PBRM1* (38 vs. 33%) presented higher somatic mutation rates in the high m6A score group, suggesting a potential explanation for the poor survival outcomes in the high m6A score group (Figures 5E, F). These findings may offer new insights for understanding the possible interactions between m6A methylation modification and somatic mutations.

### The m6A Score Correlates With TME Immune Activity and Immunotherapy

**FIGURE 6:**
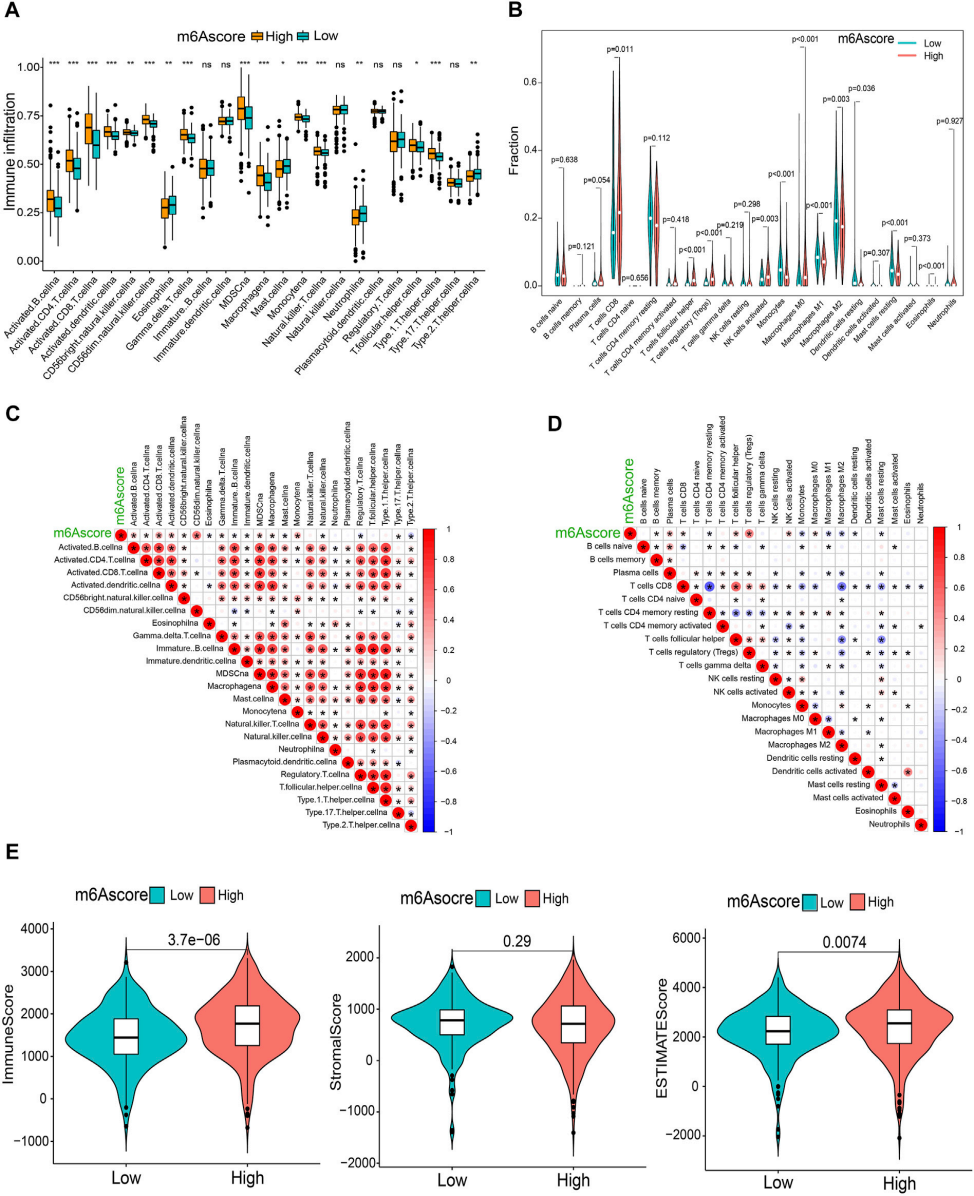
Immunological characteristics in distinct m6A score groups. **(A,B)** Differential TICs fractions between m6A score groups using sGSEA or CIBERSORT algorithm. **(C,D)** Correlations of the m6A score with TICs analyzed using ssGSEA or CIBERSORT algorithm. **(E)** Differences of TME scores between the m6A score groups. *p < 0.05; **p < 0.01; ***p < 0.001; ns, not significant.

**FIGURE 7:**
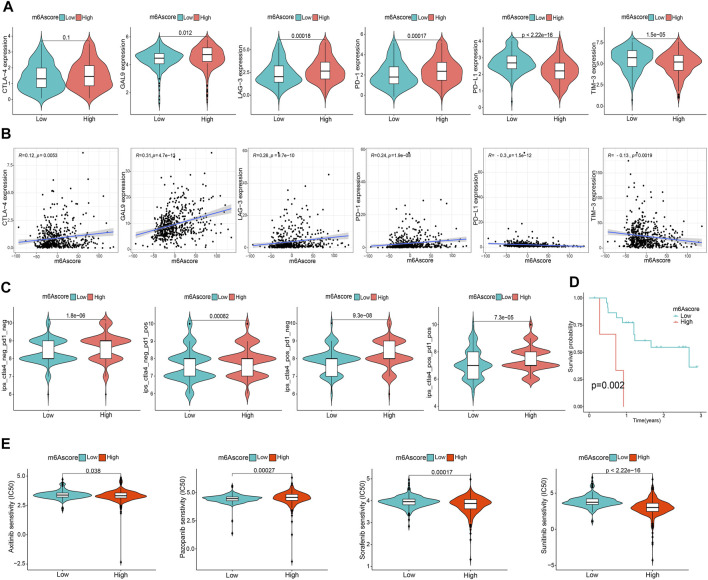
Asscociation of the m6A score with TME immune reaction. **(A)** Differential expression of six immune checkpoint genes between m6A score groups. **(B)** Correlation of m6A score with immune checkpoint-related gene expression. **(C)** Differential immunophenoscore between low and high m6A score groups. **(D)** Differential survival was compared in the GSE78220 cohort. **(E)** Differential drug sensitivity between the m6A score groups.

To comprehensively illustrate the correlation between m6A scores and TME features, we first performed difference and correlation analyses, and the results depicted that the number of most types of immune cells, such as activated CD4 T cells, activated CD8 T cells, activated dendritic cells, macrophages, natural killer T cells, follicular helper T cells, and type 1 T helper cells, was strongly associated with the m6A score (Figures 6A, C). We then validated the associations of TICs using the CIBERSORT, TIMER2.0, CIBERSORT-ABS, QUANTISEQ, MCPCOUNTER, xCell, and EPIC algorithms, and nearly all the algorithms showed that m6A score was correlated with antitumor immune cells, such as CD4 T cells and CD8 T cells (Figures 6B,D; Supplementary Figure S7). Moreover, the m6A score displayed a close association with the TME scores generated via the ESTIMATE algorithm (Figure 6E). Considering the potential immunomodulatory effects of the m6A score, we further detected the relationships between the m6A groups and six routine immune checkpoints. Notably, the expression levels of PD-L1 and TIM-3 were negatively correlated with the m6A score, which increased in the low m6A score group; GAL9, LAG-3, and PD1 expressions were positively correlated with the m6A score and were high in the high m6A score group, whereas there was no statistical difference in the CTLA-4 expression between the two groups (Figures 7A, B). Recently, immunophenoscore (IPS) has been suggested as an appreciable predictor of the responsiveness of immunotherapy (Charoentong et al., 2017). We observed that the high m6A score group experienced a higher IPS regardless of anti-PD-1/CTLA-4 therapy alone or in combination with other therapies (Figure 7C). Importantly, we verified the predicted significance of the m6A score on the response to immunotherapy in the GSE78220 cohort (Figure 7D). In addition, we compared drug sensitivity of axitinib, pazopanib, sorafenib, and sunitinib, and discovered distinct sensitivity differences in the expressions of four tyrosine kinase inhibitors (TKIs) between the high and low m6A score groups (Figure 7E).

### The Utility of the m6A Score Across Cancer Types

**FIGURE 8:**
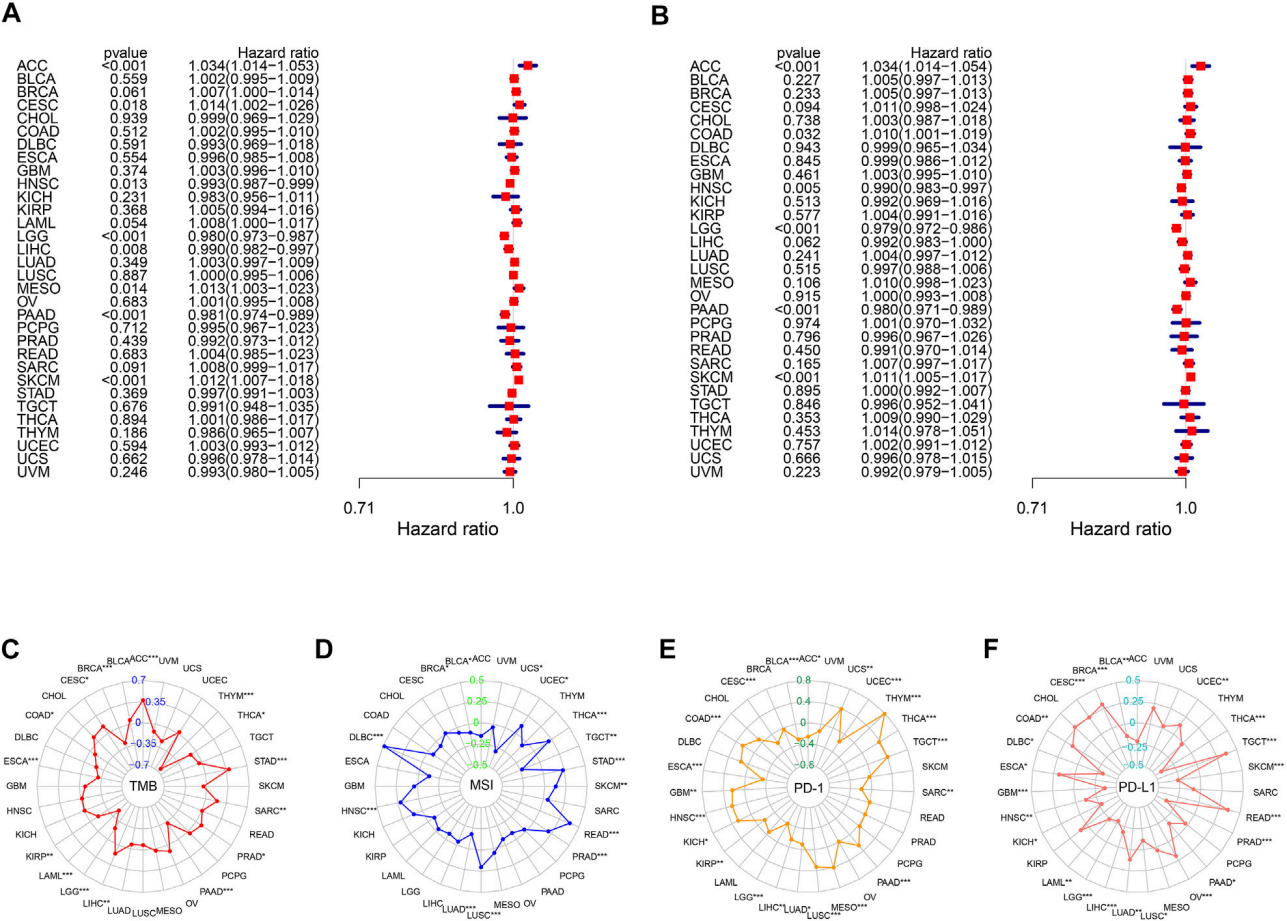
Performance of m6A score in 33 tumors. Univariable Cox regression analyses for overall survival **(A)** and disease-specific survival **(B)**. The radar graphs of correlation of m6A score with TMB **(C)**, MSI **(D)**, PD-1 and PD-L1 expression **(E,F)**. *p < 0.05; **p < 0.01; ***p < 0.001.

We further investigated the utility of the m6A score across the remaining 32 types of cancers in TCGA using the same methods used in previous analyses. Univariate Cox regression results highlighted that the m6A score was significantly related to OS in eight types of cancers (adrenocortical carcinoma (ACC), cervical squamous cell carcinoma and endocervical adenocarcinoma (CESC), head and neck squamous cell carcinoma (HNSC), LGG, LIHC, mesothelioma (MESO), PAAD, and skin cutaneous melanoma (SKCM)). We also explored the correlations between the m6A score and disease-specific survival and found that the m6A score was also a prognostic predictor in ACC, COAD, HNSC, LGG, PAAD, and SKCM (Figures 8A, B). Remarkably, Kaplan-Meier survival analysis also validated these results, reflecting the favorable predictive capacity of the m6A score (Supplementary Figures S8, 9). Moreover, we found that m6A scores were significantly correlated with TMB in 15 of 32 cancers (Figure 8C), and were related to MSI in ACC, breast invasive carcinoma (BRCA), lymphoid neoplasm diffuse large B-cell lymphoma (DLBC), HNSC, lung adenocarcinoma (LUAD), lung squamous cell carcinoma (LUSC), prostate adenocarcinoma (PRAD), rectum adenocarcinoma (READ), SKCM, STAD, testicular germ cell tumors (TGCT), thyroid carcinoma (THCA), uterine corpus endometrial carcinoma (UCEC), and uterine carcinosarcoma (UCS) (Figure 8D). We next found that there was a significant correlation between PD-1 and PD-L1 expression and the m6A score, validating the satisfactory predictive capability of the m6A score for the effect of immunotherapy (Figures 8E, F).

## Discussion

Accumulating evidence suggests that m6A modification plays pivotal roles in carcinogenesis, innate immunity, and anti-tumor immune response (He et al., 2019; Gu et al., 2021; Uddin et al., 2021). Recently, the role of m6A modification patterns in TME infiltration characterizations has been comprehensively elucidated in other solid tumors (Zhang et al., 2020; Chong et al., 2021; Du et al., 2021; Li et al., 2021; Sun et al., 2021). In this study, we explored the correlation between m6A modification and TME cell infiltration in ccRCC to enhance our apprehension of TME anti-tumor immune response and identify more effective immunotherapy strategies. Based on 23 m6A regulators, three distinct m6A modification patterns with distinct TME cell infiltration characteristics and prognoses were found. Furthermore, DEGs related to ccRCC prognosis were screened from three m6A clusters to determine three m6A-related gene signatures. Similar to the results of m6A modification clusters, these three gene signatures also had noticeably different biological functions and survival outcomes These results reveal the potential key role of m6A regulators in ccRCC. More importantly, we calculated the m6A score for each patient with ccRCC to accurately assess individual m6A modifications. Integrated analyses suggested that the m6A score is a robust and independent prognostic factor for ccRCC.

Abundant studies have emphasized that m6A modification take an active part in tumorigenesis, prognosis, and TME in ccRCC. For example, researchers have stressed that lncRNA-XIST can be modified by *METTL3* and *RBM15/15B* to promote the oncogenicity of ccRCC through the miR-302c/SDC1 axis (Patil et al., 2016; Zhang et al., 2017). Moreover, Chen et al. systematically investigated the global m6A modification pattern in ccRCC and exploited the potential correlations between abnormal m6A modifications and cancer-related gene expression (Chen et al., 2020a). In addition, several recent m6A RNA methylation regulator-based signatures have been constructed to predict the OS of ccRCC and exhibited appreciable predictive performance (Chen et al., 2020b; Ma et al., 2021; Zhang et al., 2021). However, these studies only paid attention to a few differentially expressed m6A regulators, they neither combined m6A modifications to comprehensively exploit them nor elaborated their relation to TME. Recently, Zhong et al. analyzed 513 ccRCC cases and revealed that the m6A score is an independent risk predictor that corresponds to poor prognosis of ccRCC, which also correlates with TME, consistent with our study (Zhong et al., 2021). These results indicate the vital significance of m6A modification in predicting prognosis and shaping different TME immune characteristics.

Considering the critical effect of TME on the tumorigenesis, metastasis, and immune escape in ccRCC, here we applied thorough analysis to corroborate its possible clinical significance. In the current study, infiltration of most of the anti-tumor immune cells, such as CD4 T cells, CD8 T cells, natural killer T cells, follicular helper T cells, and type 1 T helper cells, was observed in m6A cluster A and high m6A score groups. In contrast to some other types of solid tumors in which patients with cytotoxic immune cell infiltration may have a better prognosis, we noticed that patients in the high ccRCC m6A score group possessed a worse prognosis, while several critical inhibitor immune checkpoints were significantly highly expressed in the high m6A score group. Therefore, the anti-tumor effect of the high infiltration level of T cells might be inhibited by the strong immunosuppressive pathway activated by overexpressed immune checkpoints (Matsushita et al., 2016). Moreover, infiltrating CD8 T cells are reportedly involved in a dispiriting ccRCC prognosis (Fridman et al., 2017; Xiong et al., 2020).

ICIs have emerged as a promising cancer therapeutic option for advanced ccRCC (Motzer et al., 2019; Powles et al., 2020), but the immunotherapeutic outcomes exhibit individual differences because of the complexity and heterogeneity of ccRCC (Roviello et al., 2019), which indicates the clinical significance of detecting accurate predictive biomarkers of treatment efficacy. Across solid tumor malignancies, response to ICIs is reportedly associated with numerous tumor-intrinsic (such as high TMB) and TME immune-infiltrating characteristics (Yarchoan et al., 2017). We found that the m6A score was closely related to the high mutation rates of *VHL* and *PBRM1*. VHL mutation, the most common mutation in ccRCC, is positively related to PD-L1 expression and may influence the response to ccRCC anti-PD-L1/PD-1 immunotherapy (Messai et al., 2016). Furthermore, *PBRM1* mutations are significantly associated with response to anti-PD1 therapy, progression-free survival, and OS in patients with advanced ccRCC, but the predictive value is still unclear and needs to be further investigated in future larger randomized trials (Braun et al., 2019; Carril-Ajuria et al., 2019). These results indirectly indicate the underlying interplay between m6A modification and tumor somatic alterations, which may help predict immunotherapeutic outcomes. Notably, compared to the majority of other types of immunotherapy-responsive solid tumors, ccRCC has a modest mutational burden; therefore, an increasing number of studies focus on the predictive value of TICs, such as CD8 T cell proportion (Lawrence et al., 2013). In contrast to some other immune-regulated tumors that are involved in tumor infiltration along with CD8 T cells, which could drive the response to anti-PD-1 therapy, the role of CD8 T cells in response to ICIs in ccRCC remains unclear. Giraldo et al. pointed out that the activation or inhibition status of CD8 T cells could impact the efficacy of immunotherapies in ccRCC (Giraldo et al., 2017). However, Braun et al. revealed that in advanced ccRCC, immune-infiltrated phenotypes do not differ in the response to or survival after PD-1 blockade therapy in contrast to immune-deserted/excluded phenotypes (Braun et al., 2020). Moreover, tumor-associated macrophages are closely associated with the clinical benefit of anti-PD-1 treatment (Voss et al., 2018). Importantly, different m6A modification patterns have been proposed to predict immunotherapy efficacy in pan-cancers (Quan et al., 2021), here, we demonstrated the notable correlations between the m6A score and TME immune infiltration landscapes, immune checkpoint expression levels, distinct IPS, and sensitivity to TKIs, uncovering the potential usefulness of the m6A score in assessing the response to ICIs in ccRCC. Nevertheless, these results can not signify a causal correlation between m6A score and immunotherapy response in ccRCC, and we ought to gather more clinical data in future studies to validate the correlations.

When the m6A score we constructed was applied to other types of cancers, it represented poor survival accompanied by a high m6A score, which indicates that the m6A score may also reflect the aggressiveness and malignancy of cancers. Moreover, the association between immune checkpoint expression, burden of neoepitopes (TMB, MSI), and m6A score may illustrate that the phenotypes are affected by the m6A modification in tumors, contributing to uncontrolled immune disorders and dedifferentiation defined by loss of structure of origin, which was consistent with the findings of previous studies (Zhang et al., 2020; Chong et al., 2021; Du et al., 2021; Li et al., 2021; Sun et al., 2021), indicating the robust and potential predictive value of the m6A score. However, this study has some limitations. First, although we enrolled 23 generally approved m6A regulators in the previous studies, it is necessary to incorporate some newly identified m6A regulators into the signature to improve the accuracy and reliability of our results. Second, we gathered data from TCGA and GEO to expand the sample size and verify our results, so there may exist unavoidable errors in the process of integrating data. In addition, given that the study lacked high numbers of ccRCC samples and independent clinical cohorts, we can not clinically verify the findings. Thus, further validation based on prospective cohorts of patients with ccRCC receiving immunotherapy is required in the future.

In conclusion, this study elucidated a correlation between m6A modification patterns and TME immune-infiltrating characteristics in ccRCC. A comprehensive assessment of the m6A modification pattern in ccRCC will be useful in understanding the TME immune-infiltrating characteristics and in the determination of immunotherapy strategies.

## Data Availability

The datasets presented in this study can be found in online repositories. The names of the repository/repositories and accession number(s) can be found in the article/Supplementary Material.
