# Supplementary material for: m6A Methylation Patterns and Tumor Microenvironment Infiltration Characterization in Clear-Cell Renal Cell Carcinoma
Source: Front Genet. 2022 Apr 21;13:864549. doi: 10.3389/fgene.2022.864549 (PMC9068873; doi:10.3389/fgene.2022.864549)
Supplement: Supplementary file 1 [file DataSheet1.DOCX]

Supplementary Material

# Supplementary Figures and Tables

## Supplementary Figures


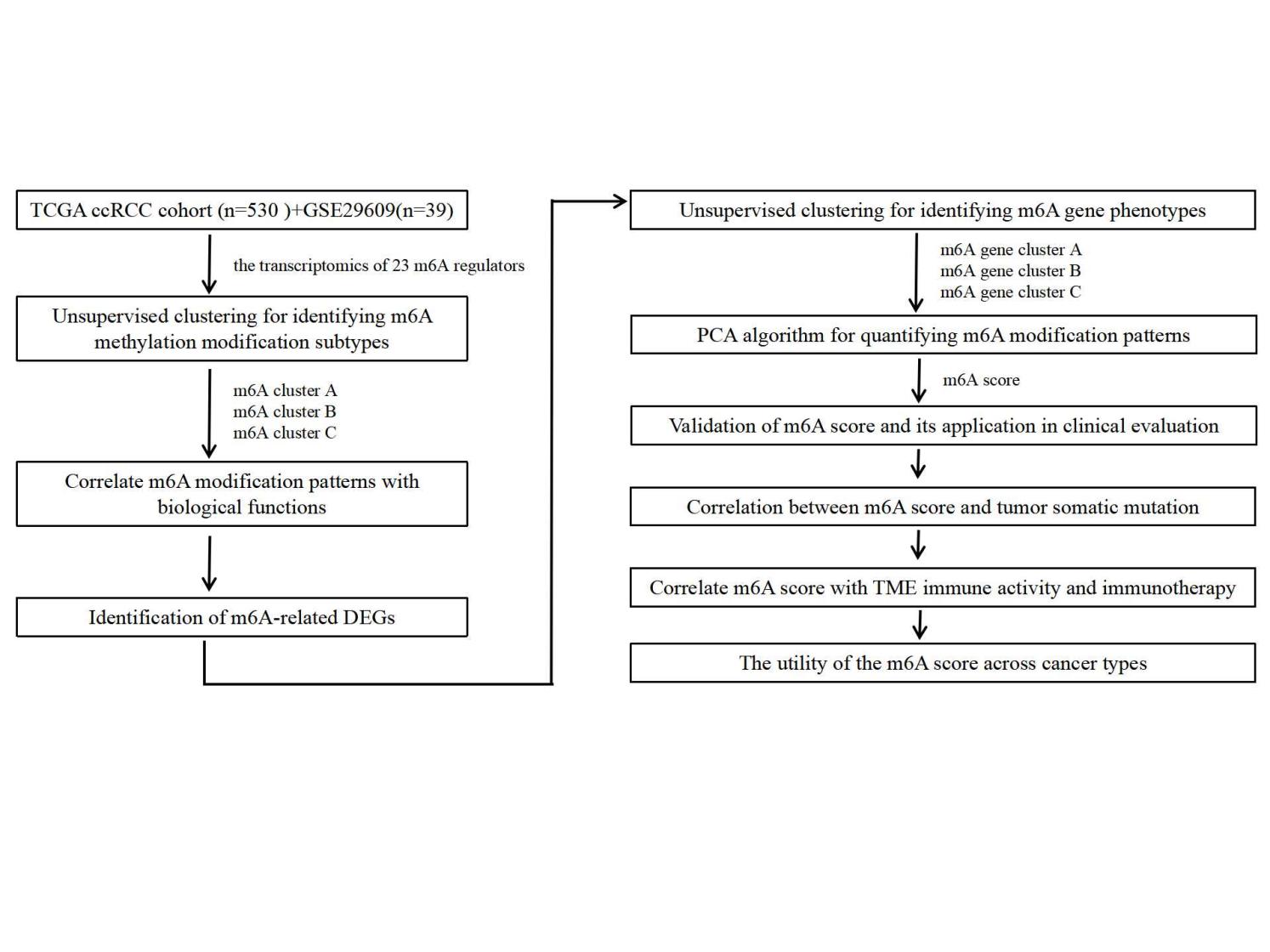


**Figure S1**. The flow chart of this study.


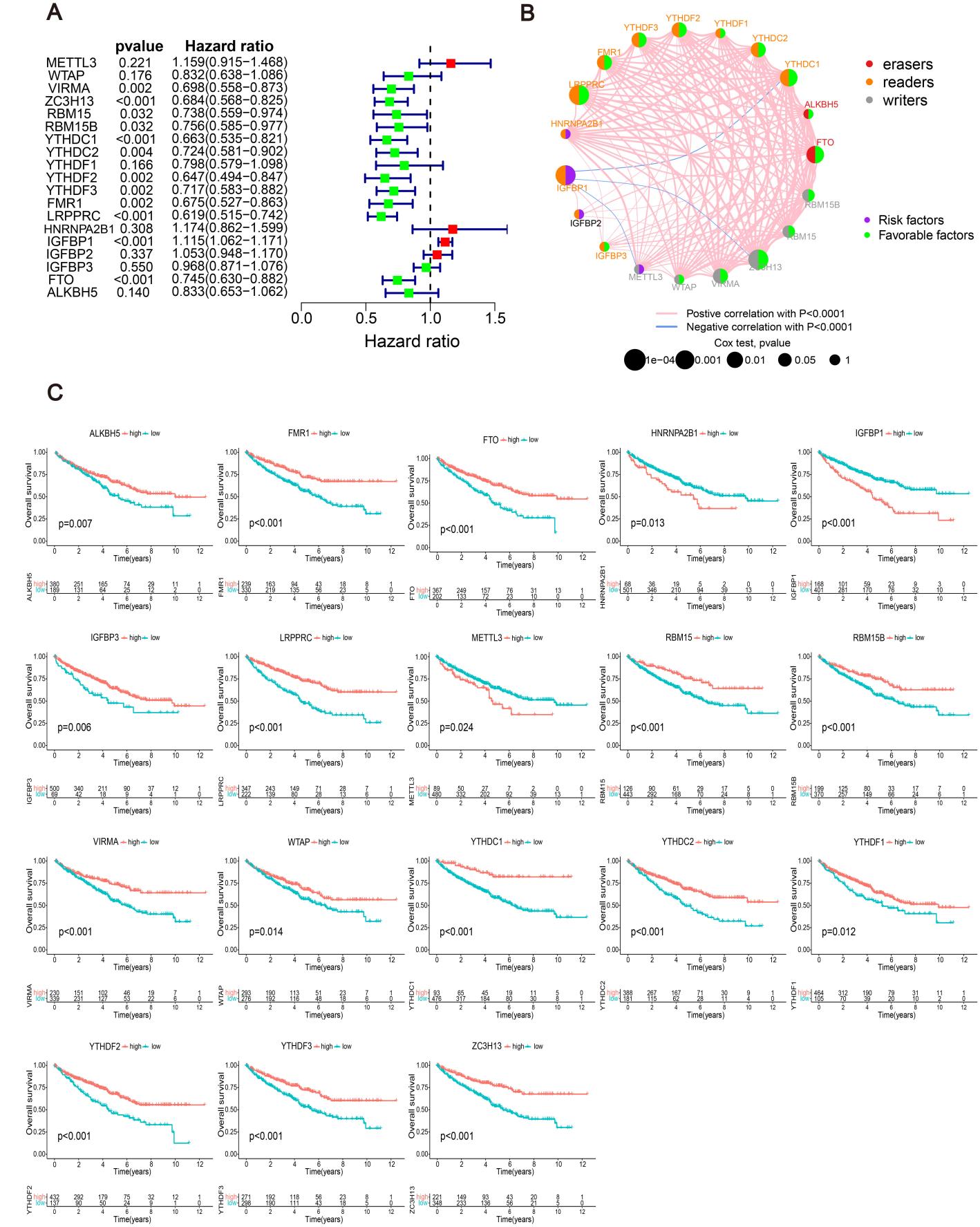


**Figure S2.** Survival and correlation analysis of RNA N6-methyladenosine (m6A) regulators. (A) Forest plots of m6A regulators were generated using univariate Cox regression. (B) Correlations between m6A regulators in clear-cell renal cell carcinoma. (C) The survival curves of m6A regulators were calculated using K-M methods.

**
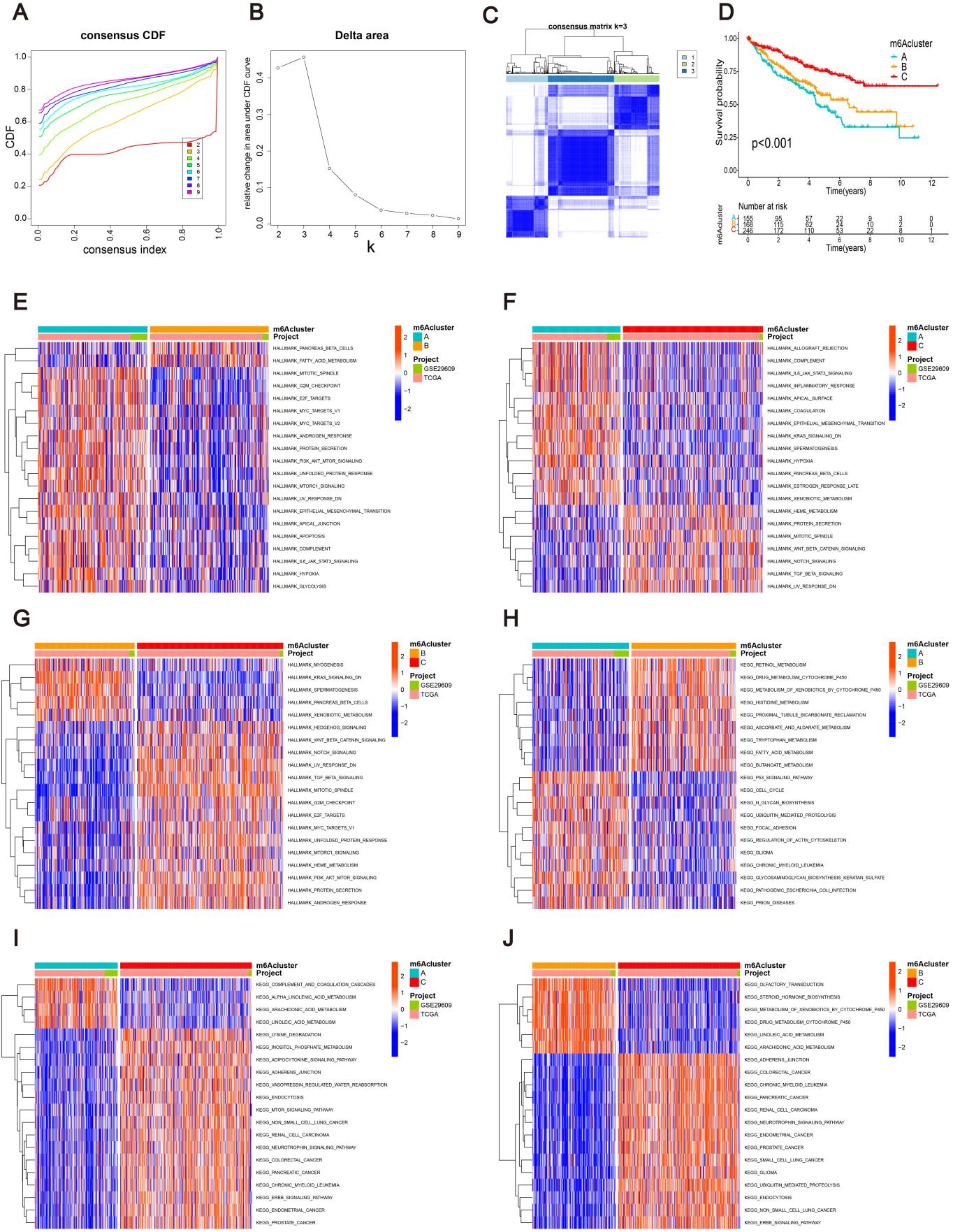
**

**Figure S3.** Identification of m6A clusters. (A) Consensus clustering cumulative distribution function for k=2 to 9. (B) Relative change in the area under the cumulative distribution function curve for k=2 to 9. (C) Consensus matrix for k=3. (D) Kaplan-Meier survival analysis for the three m6A clusters. (E-J) Gene set variation analysis for the m6A clusters.

**
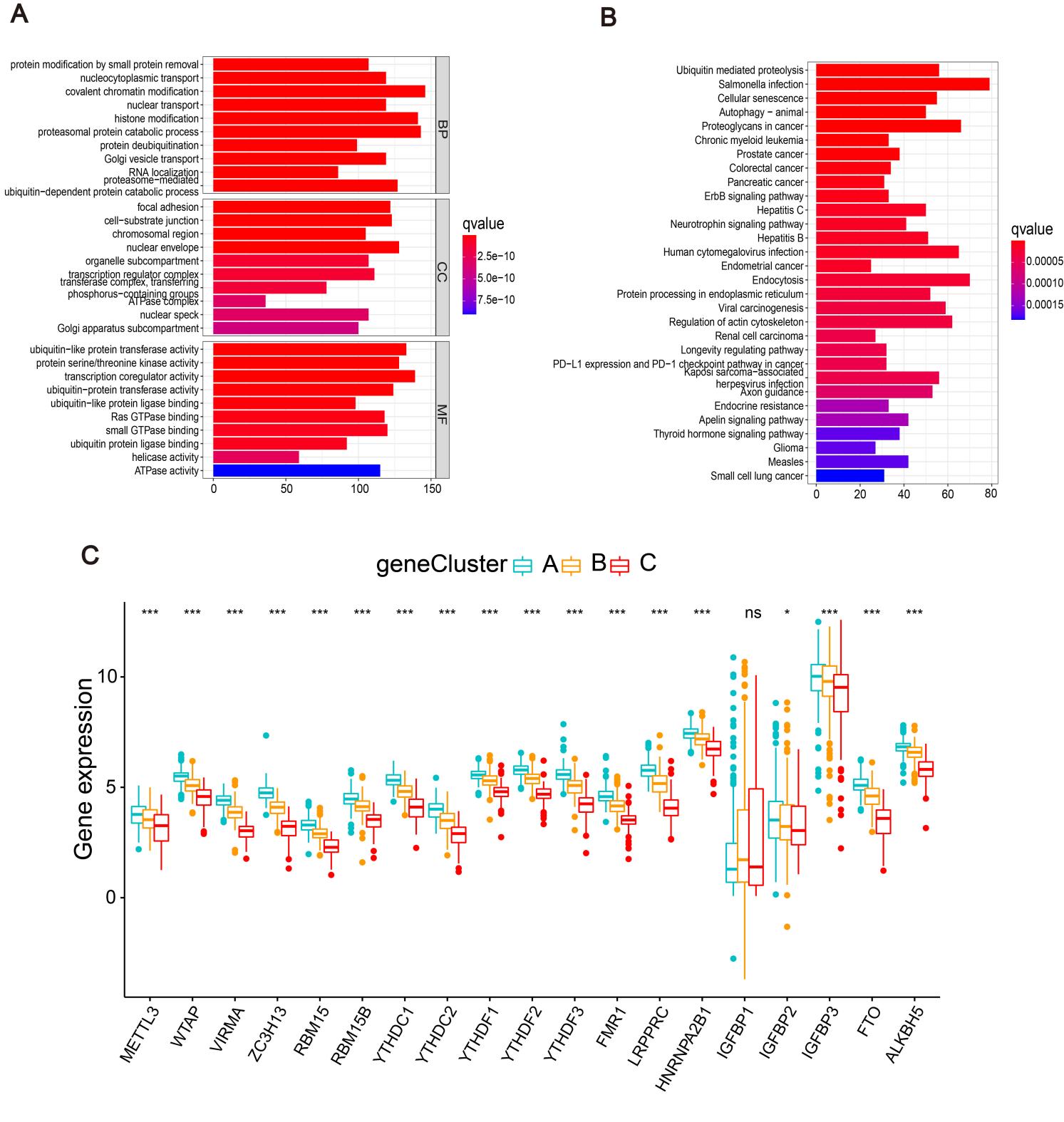
Figure S4.** Functional annotation for m6A signature -related genes.(A,B) Gene ontology and Kyoto Encyclopedia of Genes and Genomes enrichment analysis of differentially expressed genes determined from three m6A clusters. (C) Differential expression of m6A regulators between distinct gene clusters. *p < 0.05, **p < 0.01, ***p < 0.001, ns, not significant.

**
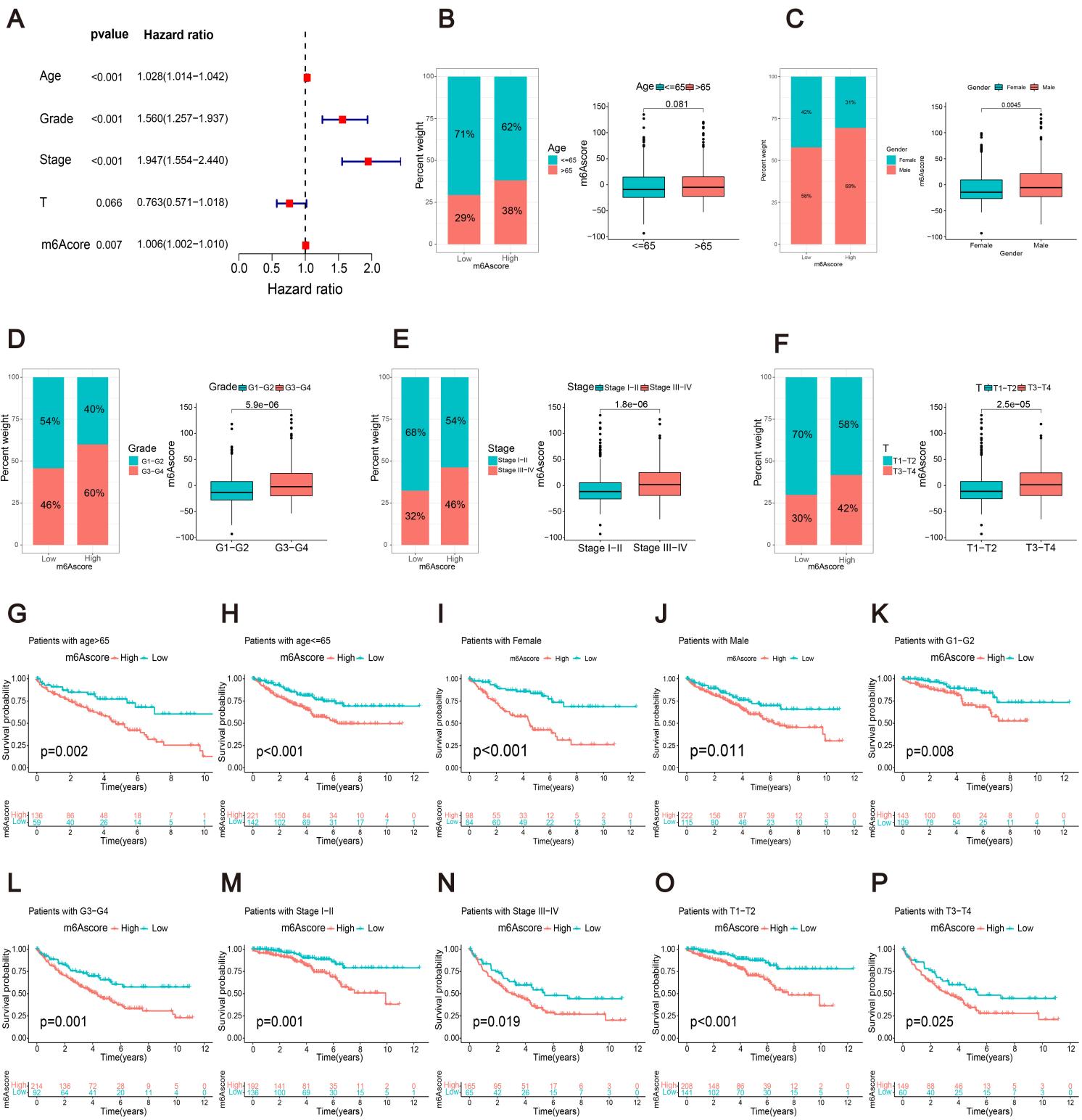
**

**Figure S5.** Validation of the m6A score. (A) Multivariate Cox regression analysis validated the m6A score as an independent prognostic indicator. Distribution of m6A score stratified by age (B), sex (C), grade (D), stage (E), and T stage (F). Analysis of overall survival stratified by age (G,H), sex (I,J), grade (K,L), stage (M,N), and T stage (O,P).

**
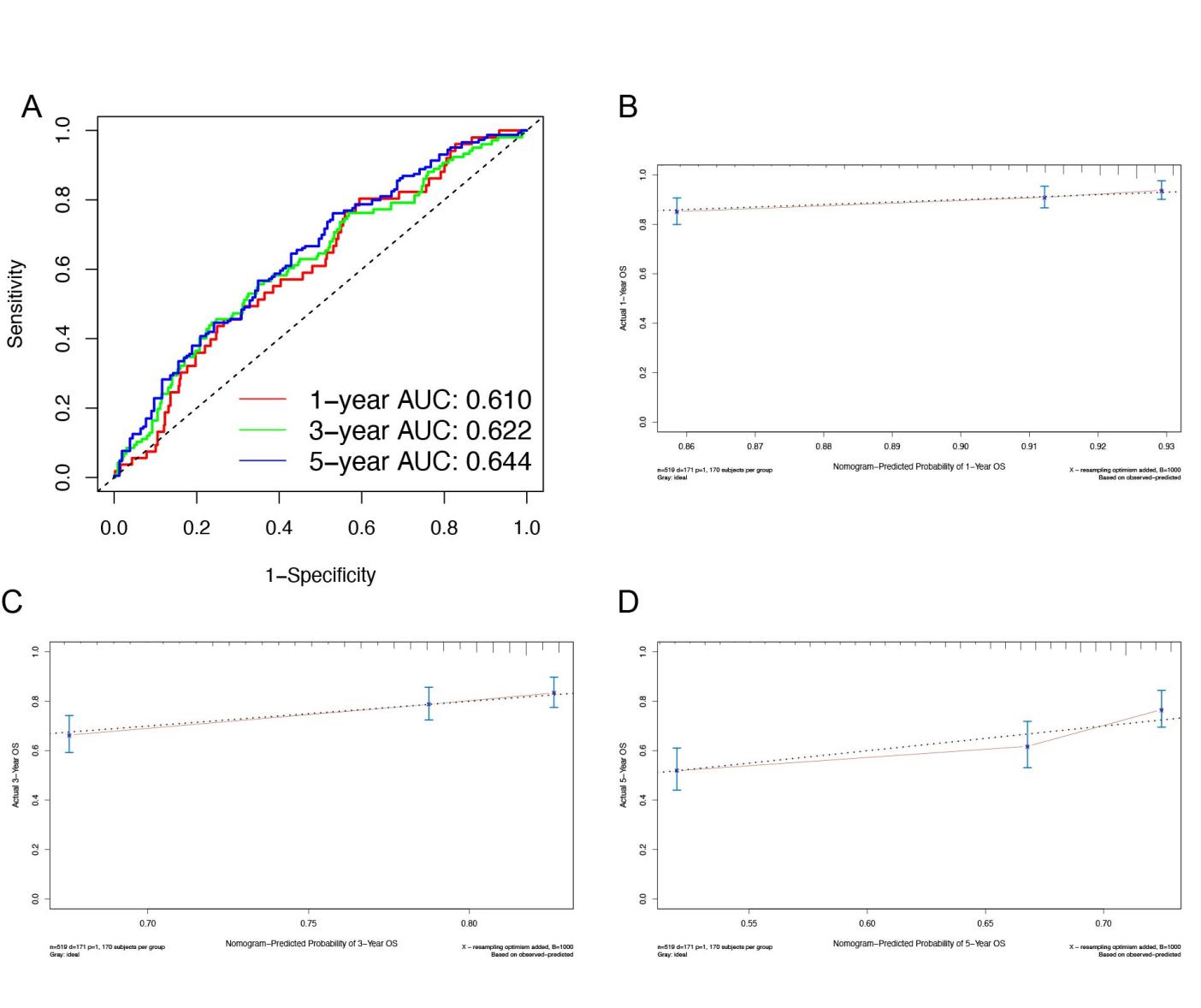
**

**Figure S6.** (A) ROC curves of m6A score for predicting 1-, 3-, and 5-years OS rates in ccRCC cohort. (B-D) Calibration curves of m6A score for predicting the probability of OS at 1-, 3- and 5- years (bootstrap method, 1,000 repetitions).

**
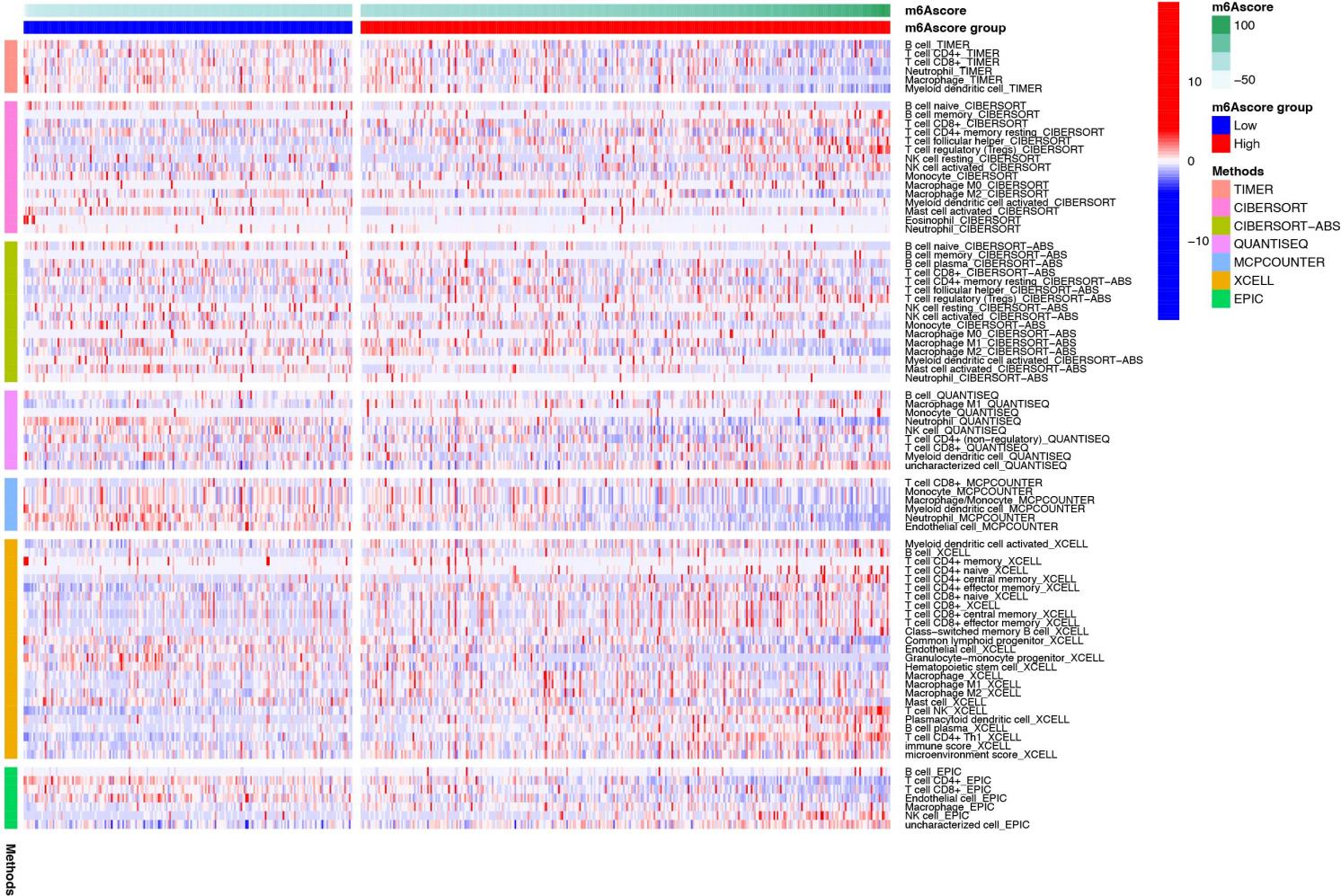
**

**Figure S7.** The heatmap of the immune cells inflitration determined by various algorithms.

**
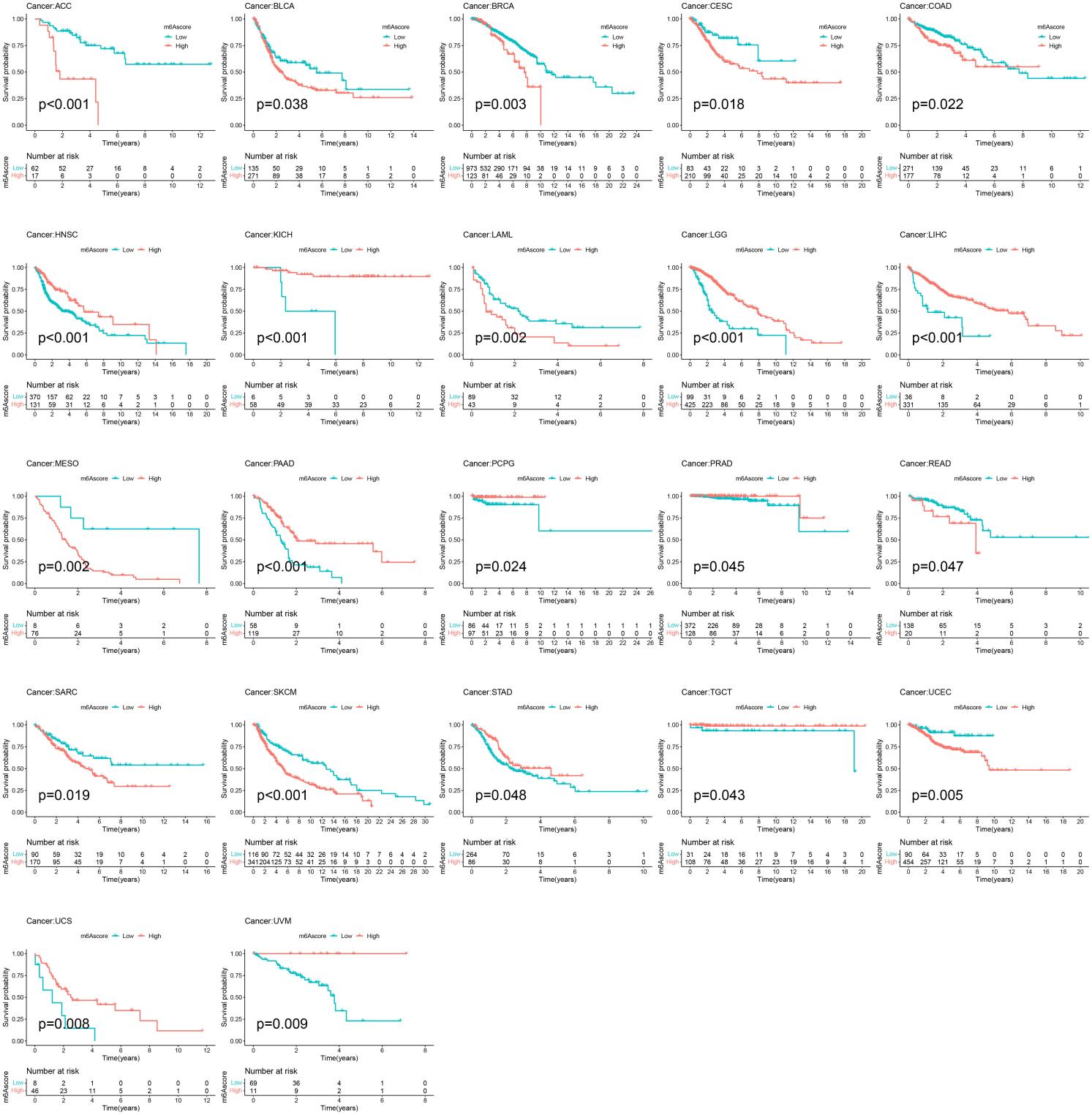
**

**Figure S8.** Kaplan-Meier curves indicating the relationship between the m6A score and overall survival in 33 tumors.

**
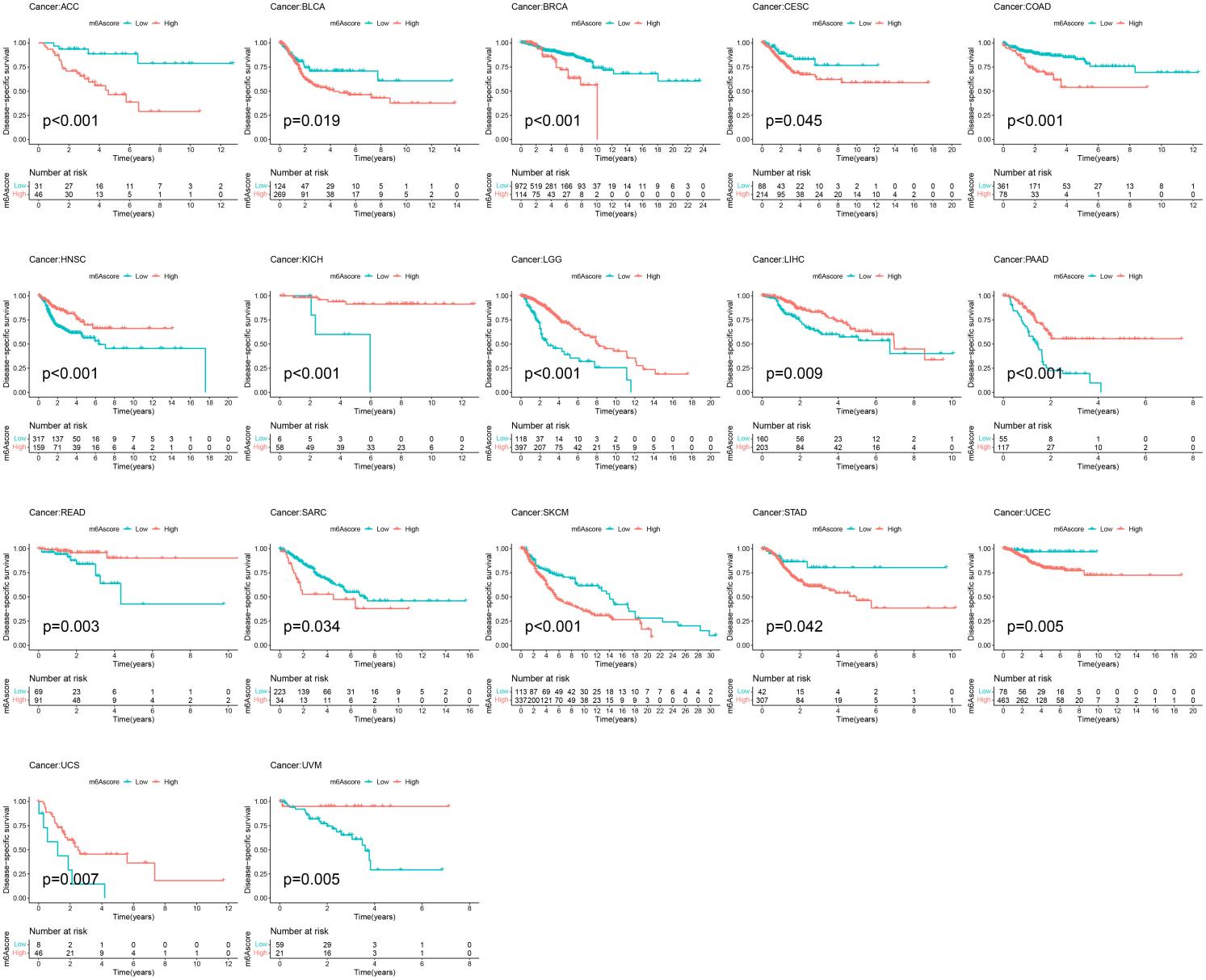
**

**Figure S9.** Kaplan-Meier curves indicating the relationship between the m6A score and disease specific survival in 33 tumors.

## Supplementary Tables

**Table S1.** Dataset information included in this study.

| **Accession Number** | **GPL** | **Platform** | **%Male** | **Age (mean±sd)** | **Grade (G1/G2/G3/G4)** | **Stage(I/II/III/IV)** | **Number of  eligible renal cell carcinoma Patients / Samples** | **Survival Data** |
| --- | --- | --- | --- | --- | --- | --- | --- | --- |
| GSE22541 | GPL570 | Affymetrix Human Genome U133 Plus 2.0 Array | 54.17 | NA | NA/18/6/NA | NA/7/5/4 | 24 | Disease-free survival |
| GSE29609 | GPL1708 | Agilent-012391 Whole Human Genome Oligo Microarray G4112A | NA | 61.38±12.78 | 1/12/11/15 | 10/3/12/14 | 39 | Overall survival |
| GSE78220 | GPL11154 | Illumina HiSeq 2000 (Homo sapiens) | 69.23 | 59.27±15.06 | NA | NA | 26 | Overall survival |
| TCGA-KIRC | - | Illumina HiSeq 2000 RNA Sequencing V2 | 64.91 | 60.56±12.14 | NA/227/206/75 | NA/57/123/82 | 530 | Overall survival |

**Table S2.** Prognostic analysis of m6A genes.

| **m6A genes** | **HR** | **HR.95L** | **HR.95H** | **P value** | **km** |
| --- | --- | --- | --- | --- | --- |
| METTL3 | 1.159171119 | 0.915156275 | 1.468249434 | 0.22065658 | 0.023888841 |
| WTAP | 0.832407278 | 0.638288435 | 1.085562324 | 0.175744548 | 0.013837302 |
| VIRMA | 0.698001503 | 0.558077274 | 0.873008309 | 0.001634111 | 0.000142071 |
| ZC3H13 | 0.684355841 | 0.567876529 | 0.824726666 | 6.77E-05 | 1.03E-06 |
| RBM15 | 0.737796982 | 0.558805055 | 0.97412216 | 0.031960949 | 0.000853519 |
| RBM15B | 0.756161453 | 0.585452708 | 0.976646166 | 0.032275919 | 0.000978964 |
| YTHDC1 | 0.663005566 | 0.535395504 | 0.821031139 | 0.000164635 | 1.31E-05 |
| YTHDC2 | 0.724235505 | 0.581316755 | 0.902291327 | 0.004018483 | 1.14E-05 |
| YTHDF1 | 0.79751151 | 0.579019269 | 1.09845154 | 0.166016932 | 0.01207478 |
| YTHDF2 | 0.647157554 | 0.494299283 | 0.847286077 | 0.001548776 | 1.10E-05 |
| YTHDF3 | 0.716806345 | 0.582777659 | 0.881659288 | 0.001618742 | 2.23E-05 |
| FMR1 | 0.674663434 | 0.527323453 | 0.863171828 | 0.001745761 | 1.45E-05 |
| LRPPRC | 0.618583623 | 0.515479792 | 0.742309796 | 2.43E-07 | 3.47E-09 |
| HNRNPA2B1 | 1.174289861 | 0.862331952 | 1.599101918 | 0.307819547 | 0.012982553 |
| IGFBP1 | 1.114997524 | 1.061942182 | 1.170703546 | 1.21E-05 | 9.89E-08 |
| IGFBP2 | 1.052893507 | 0.947751884 | 1.169699323 | 0.336939442 | 0.111881918 |
| IGFBP3 | 0.96827572 | 0.871198338 | 1.076170406 | 0.549782694 | 0.006047437 |
| FTO | 0.745306554 | 0.629634628 | 0.88222889 | 0.000635182 | 1.91E-06 |
| ALKBH5 | 0.832518913 | 0.652692983 | 1.061889371 | 0.139859132 | 0.006787384 |

**Table S3.** Spearman correlation analysis of the m6A modification regulators.

| **From** | **To** | **Cor** | **P value** | **Weight** |
| --- | --- | --- | --- | --- |
| FTO | ALKBH5 | 0.679185 | 3.39E-78 | 4.075113 |
| FTO | YTHDC1 | 0.706420 | 3.73E-87 | 4.238519 |
| FTO | YTHDC2 | 0.654425 | 7.44E-71 | 3.926550 |
| FTO | YTHDF1 | 0.501081 | 1.68E-37 | 3.006485 |
| FTO | YTHDF2 | 0.644133 | 5.29E-68 | 3.864795 |
| FTO | YTHDF3 | 0.552347 | 9.29E-47 | 3.314083 |
| FTO | FMR1 | 0.500952 | 1.76E-37 | 3.005712 |
| FTO | LRPPRC | 0.626714 | 2.03E-63 | 3.760283 |
| FTO | HNRNPA2B1 | 0.436211 | 7.85E-28 | 2.617266 |
| FTO | IGFBP3 | 0.381842 | 3.43E-21 | 2.291049 |
| FTO | METTL3 | 0.222546 | 8.13E-08 | 1.335275 |
| FTO | WTAP | 0.578464 | 3.99E-52 | 3.470782 |
| FTO | VIRMA | 0.676035 | 3.20E-77 | 4.056211 |
| FTO | ZC3H13 | 0.678501 | 5.53E-78 | 4.071008 |
| FTO | RBM15 | 0.633830 | 2.95E-65 | 3.802980 |
| FTO | RBM15B | 0.494770 | 1.82E-36 | 2.968621 |
| ALKBH5 | YTHDC1 | 0.651354 | 5.42E-70 | 3.908122 |
| ALKBH5 | YTHDC2 | 0.468419 | 2.25E-32 | 2.810513 |
| ALKBH5 | YTHDF1 | 0.649893 | 1.38E-69 | 3.899360 |
| ALKBH5 | YTHDF2 | 0.687645 | 7.14E-81 | 4.125872 |
| ALKBH5 | YTHDF3 | 0.455451 | 1.74E-30 | 2.732707 |
| ALKBH5 | FMR1 | 0.420723 | 8.17E-26 | 2.524336 |
| ALKBH5 | LRPPRC | 0.509893 | 5.55E-39 | 3.059356 |
| ALKBH5 | HNRNPA2B1 | 0.515960 | 5.00E-40 | 3.095759 |
| ALKBH5 | IGFBP2 | 0.163594 | 8.85E-05 | 0.981561 |
| ALKBH5 | IGFBP3 | 0.339901 | 7.45E-17 | 2.039406 |
| ALKBH5 | METTL3 | 0.349913 | 7.85E-18 | 2.099476 |
| ALKBH5 | WTAP | 0.497522 | 6.47E-37 | 2.985131 |
| ALKBH5 | VIRMA | 0.582771 | 4.67E-53 | 3.496626 |
| ALKBH5 | ZC3H13 | 0.596958 | 3.15E-56 | 3.581746 |
| ALKBH5 | RBM15 | 0.637634 | 2.94E-66 | 3.825806 |
| ALKBH5 | RBM15B | 0.621054 | 5.43E-62 | 3.726323 |
| YTHDC1 | YTHDC2 | 0.681957 | 4.61E-79 | 4.091742 |
| YTHDC1 | YTHDF1 | 0.651400 | 5.26E-70 | 3.908399 |
| YTHDC1 | YTHDF2 | 0.687863 | 6.08E-81 | 4.127178 |
| YTHDC1 | YTHDF3 | 0.618204 | 2.77E-61 | 3.709222 |
| YTHDC1 | FMR1 | 0.682629 | 2.83E-79 | 4.095772 |
| YTHDC1 | LRPPRC | 0.652501 | 2.59E-70 | 3.915009 |
| YTHDC1 | HNRNPA2B1 | 0.705312 | 9.05E-87 | 4.231869 |
| YTHDC1 | IGFBP1 | -0.260259 | 2.91E-10 | 1.561554 |
| YTHDC1 | IGFBP3 | 0.272023 | 4.13E-11 | 1.632137 |
| YTHDC1 | METTL3 | 0.557630 | 8.33E-48 | 3.345781 |
| YTHDC1 | WTAP | 0.620363 | 8.07E-62 | 3.722181 |
| YTHDC1 | VIRMA | 0.716056 | 1.41E-90 | 4.296339 |
| YTHDC1 | ZC3H13 | 0.806048 | 2.90E-131 | 4.836288 |
| YTHDC1 | RBM15 | 0.717218 | 5.35E-91 | 4.303309 |
| YTHDC1 | RBM15B | 0.610873 | 1.70E-59 | 3.665237 |
| YTHDC2 | YTHDF1 | 0.508934 | 8.08E-39 | 3.053605 |
| YTHDC2 | YTHDF2 | 0.592575 | 3.12E-55 | 3.555452 |
| YTHDC2 | YTHDF3 | 0.604718 | 4.96E-58 | 3.628307 |
| YTHDC2 | FMR1 | 0.675289 | 5.41E-77 | 4.051733 |
| YTHDC2 | LRPPRC | 0.583610 | 3.06E-53 | 3.501660 |
| YTHDC2 | HNRNPA2B1 | 0.622307 | 2.64E-62 | 3.733841 |
| YTHDC2 | IGFBP3 | 0.252840 | 9.50E-10 | 1.517042 |
| YTHDC2 | METTL3 | 0.414338 | 5.17E-25 | 2.486026 |
| YTHDC2 | WTAP | 0.598614 | 1.31E-56 | 3.591684 |
| YTHDC2 | VIRMA | 0.617560 | 4.00E-61 | 3.705361 |
| YTHDC2 | ZC3H13 | 0.664350 | 1.03E-73 | 3.986101 |
| YTHDC2 | RBM15 | 0.619699 | 1.18E-61 | 3.718191 |
| YTHDC2 | RBM15B | 0.387756 | 7.43E-22 | 2.326538 |
| YTHDF1 | YTHDF2 | 0.711699 | 5.19E-89 | 4.270194 |
| YTHDF1 | YTHDF3 | 0.599279 | 9.21E-57 | 3.595672 |
| YTHDF1 | FMR1 | 0.535727 | 1.39E-43 | 3.214365 |
| YTHDF1 | LRPPRC | 0.524192 | 1.77E-41 | 3.145150 |
| YTHDF1 | HNRNPA2B1 | 0.671361 | 8.46E-76 | 4.028165 |
| YTHDF1 | IGFBP2 | 0.189105 | 5.57E-06 | 1.134632 |
| YTHDF1 | METTL3 | 0.405833 | 5.69E-24 | 2.435000 |
| YTHDF1 | WTAP | 0.571276 | 1.34E-50 | 3.427654 |
| YTHDF1 | VIRMA | 0.547711 | 7.45E-46 | 3.286263 |
| YTHDF1 | ZC3H13 | 0.612800 | 5.82E-60 | 3.676800 |
| YTHDF1 | RBM15 | 0.607916 | 8.68E-59 | 3.647496 |
| YTHDF1 | RBM15B | 0.702341 | 9.52E-86 | 4.214046 |
| YTHDF2 | YTHDF3 | 0.705842 | 5.92E-87 | 4.235054 |
| YTHDF2 | FMR1 | 0.556294 | 1.54E-47 | 3.337766 |
| YTHDF2 | LRPPRC | 0.615363 | 1.38E-60 | 3.692180 |
| YTHDF2 | HNRNPA2B1 | 0.536419 | 1.04E-43 | 3.218515 |
| YTHDF2 | IGFBP3 | 0.187362 | 6.82E-06 | 1.124171 |
| YTHDF2 | METTL3 | 0.260787 | 2.67E-10 | 1.564723 |
| YTHDF2 | WTAP | 0.663626 | 1.68E-73 | 3.981755 |
| YTHDF2 | VIRMA | 0.705379 | 8.58E-87 | 4.232274 |
| YTHDF2 | ZC3H13 | 0.703592 | 3.55E-86 | 4.221554 |
| YTHDF2 | RBM15 | 0.732793 | 7.17E-97 | 4.396758 |
| YTHDF2 | RBM15B | 0.673335 | 2.14E-76 | 4.040010 |
| YTHDF3 | FMR1 | 0.719390 | 8.57E-92 | 4.316343 |
| YTHDF3 | LRPPRC | 0.737889 | 6.97E-99 | 4.427337 |
| YTHDF3 | HNRNPA2B1 | 0.473334 | 4.13E-33 | 2.840005 |
| YTHDF3 | METTL3 | 0.231490 | 2.33E-08 | 1.388937 |
| YTHDF3 | WTAP | 0.588869 | 2.12E-54 | 3.533212 |
| YTHDF3 | VIRMA | 0.798859 | 2.86E-127 | 4.793154 |
| YTHDF3 | ZC3H13 | 0.713383 | 1.30E-89 | 4.280300 |
| YTHDF3 | RBM15 | 0.609939 | 2.85E-59 | 3.659632 |
| YTHDF3 | RBM15B | 0.525789 | 9.14E-42 | 3.154736 |
| FMR1 | LRPPRC | 0.668529 | 5.98E-75 | 4.011177 |
| FMR1 | HNRNPA2B1 | 0.553436 | 5.67E-47 | 3.320618 |
| FMR1 | METTL3 | 0.422573 | 4.75E-26 | 2.535435 |
| FMR1 | WTAP | 0.537781 | 5.77E-44 | 3.226687 |
| FMR1 | VIRMA | 0.685883 | 2.62E-80 | 4.115301 |
| FMR1 | ZC3H13 | 0.717473 | 4.32E-91 | 4.304840 |
| FMR1 | RBM15 | 0.533721 | 3.28E-43 | 3.202327 |
| FMR1 | RBM15B | 0.502039 | 1.16E-37 | 3.012237 |
| LRPPRC | HNRNPA2B1 | 0.384208 | 1.87E-21 | 2.305249 |
| LRPPRC | METTL3 | 0.239851 | 6.89E-09 | 1.439104 |
| LRPPRC | WTAP | 0.524024 | 1.89E-41 | 3.144142 |
| LRPPRC | VIRMA | 0.757999 | 2.63E-107 | 4.547993 |
| LRPPRC | ZC3H13 | 0.727723 | 6.48E-95 | 4.366340 |
| LRPPRC | RBM15 | 0.565805 | 1.83E-49 | 3.394833 |
| LRPPRC | RBM15B | 0.536785 | 8.86E-44 | 3.220707 |
| HNRNPA2B1 | IGFBP3 | 0.170092 | 4.54E-05 | 1.020552 |
| HNRNPA2B1 | METTL3 | 0.669405 | 3.27E-75 | 4.016430 |
| HNRNPA2B1 | WTAP | 0.589650 | 1.42E-54 | 3.537903 |
| HNRNPA2B1 | VIRMA | 0.486576 | 3.72E-35 | 2.919454 |
| HNRNPA2B1 | ZC3H13 | 0.642505 | 1.46E-67 | 3.855029 |
| HNRNPA2B1 | RBM15 | 0.585165 | 1.40E-53 | 3.510991 |
| HNRNPA2B1 | RBM15B | 0.570834 | 1.66E-50 | 3.425006 |
| IGFBP1 | IGFBP2 | 0.233106 | 1.85E-08 | 1.398638 |
| IGFBP1 | IGFBP3 | 0.246538 | 2.52E-09 | 1.479226 |
| IGFBP1 | METTL3 | -0.185277 | 8.65E-06 | 1.111661 |
| IGFBP1 | ZC3H13 | -0.181655 | 1.30E-05 | 1.089932 |
| IGFBP2 | VIRMA | 0.175402 | 2.58E-05 | 1.052410 |
| IGFBP2 | RBM15B | 0.363115 | 3.56E-19 | 2.178691 |
| IGFBP3 | WTAP | 0.226765 | 4.53E-08 | 1.360588 |
| IGFBP3 | VIRMA | 0.219251 | 1.27E-07 | 1.315508 |
| IGFBP3 | ZC3H13 | 0.175008 | 2.69E-05 | 1.050046 |
| IGFBP3 | RBM15 | 0.245280 | 3.05E-09 | 1.471678 |
| METTL3 | WTAP | 0.339353 | 8.41E-17 | 2.036116 |
| METTL3 | VIRMA | 0.285958 | 3.61E-12 | 1.715747 |
| METTL3 | ZC3H13 | 0.406137 | 5.23E-24 | 2.436823 |
| METTL3 | RBM15 | 0.427591 | 1.07E-26 | 2.565547 |
| METTL3 | RBM15B | 0.393444 | 1.66E-22 | 2.360666 |
| WTAP | VIRMA | 0.575474 | 1.74E-51 | 3.452842 |
| WTAP | ZC3H13 | 0.578433 | 4.05E-52 | 3.470600 |
| WTAP | RBM15 | 0.542707 | 6.79E-45 | 3.256243 |
| WTAP | RBM15B | 0.490561 | 8.65E-36 | 2.943366 |
| VIRMA | ZC3H13 | 0.788913 | 5.27E-122 | 4.733477 |
| VIRMA | RBM15 | 0.618171 | 2.82E-61 | 3.709024 |
| VIRMA | RBM15B | 0.617141 | 5.07E-61 | 3.702846 |
| ZC3H13 | RBM15 | 0.654376 | 7.68E-71 | 3.926254 |
| ZC3H13 | RBM15B | 0.698809 | 1.50E-84 | 4.192856 |
| RBM15 | RBM15B | 0.594960 | 9.00E-56 | 3.569761 |

**Table S4.** Determination of three m6A modification patterns.

| **ID** | **m6Acluster** |
| --- | --- |
| TCGA_TCGA-CJ-4920 | A |
| TCGA_TCGA-BP-5177 | B |
| TCGA_TCGA-B0-4842 | B |
| TCGA_TCGA-BP-4343 | A |
| TCGA_TCGA-MW-A4EC | C |
| TCGA_TCGA-B8-4143 | A |
| TCGA_TCGA-CJ-5675 | C |
| TCGA_TCGA-BP-4760 | C |
| TCGA_TCGA-B0-5113 | C |
| TCGA_TCGA-BP-4340 | B |
| TCGA_TCGA-B8-A7U6 | A |
| TCGA_TCGA-B8-5551 | A |
| TCGA_TCGA-B0-5400 | A |
| TCGA_TCGA-BP-4994 | B |
| TCGA_TCGA-A3-3349 | C |
| TCGA_TCGA-AK-3455 | A |
| TCGA_TCGA-CW-5585 | C |
| TCGA_TCGA-BP-4981 | B |
| TCGA_TCGA-BP-4337 | A |
| TCGA_TCGA-CJ-5686 | A |
| TCGA_TCGA-A3-A8OV | B |
| TCGA_TCGA-AK-3447 | B |
| TCGA_TCGA-B0-5702 | B |
| TCGA_TCGA-CJ-4918 | A |
| TCGA_TCGA-B0-4848 | C |
| TCGA_TCGA-BP-5194 | C |
| TCGA_TCGA-B2-5635 | C |
| TCGA_TCGA-B0-5096 | A |
| TCGA_TCGA-CZ-5986 | C |
| TCGA_TCGA-A3-3380 | A |
| TCGA_TCGA-BP-4329 | B |
| TCGA_TCGA-B0-4712 | A |
| TCGA_TCGA-B0-4691 | B |
| TCGA_TCGA-CZ-5982 | C |
| TCGA_TCGA-BP-4962 | B |
| TCGA_TCGA-CZ-5466 | B |
| TCGA_TCGA-AK-3450 | C |
| TCGA_TCGA-CJ-4876 | B |
| TCGA_TCGA-B0-4834 | A |
| TCGA_TCGA-B0-5713 | C |
| TCGA_TCGA-BP-5009 | B |
| TCGA_TCGA-CJ-4900 | A |
| TCGA_TCGA-BP-5186 | C |
| TCGA_TCGA-A3-3370 | C |
| TCGA_TCGA-B0-4810 | A |
| TCGA_TCGA-CJ-4916 | B |
| TCGA_TCGA-B0-4846 | C |
| TCGA_TCGA-CJ-4638 | B |
| TCGA_TCGA-BP-5184 | C |
| TCGA_TCGA-B0-5116 | C |
| TCGA_TCGA-CZ-4857 | A |
| TCGA_TCGA-B0-4718 | B |
| TCGA_TCGA-B0-5696 | B |
| TCGA_TCGA-BP-4326 | A |
| TCGA_TCGA-CZ-4856 | C |
| TCGA_TCGA-BP-4972 | C |
| TCGA_TCGA-BP-5169 | A |
| TCGA_TCGA-B2-5633 | C |
| TCGA_TCGA-B0-5693 | C |
| TCGA_TCGA-BP-4969 | B |
| TCGA_TCGA-BP-4165 | B |
| TCGA_TCGA-CJ-4870 | B |
| TCGA_TCGA-B8-5552 | C |
| TCGA_TCGA-BP-4164 | C |
| TCGA_TCGA-BP-4987 | C |
| TCGA_TCGA-BP-4769 | C |
| TCGA_TCGA-BP-4161 | A |
| TCGA_TCGA-BP-5010 | B |
| TCGA_TCGA-B2-3923 | B |
| TCGA_TCGA-AK-3445 | A |
| TCGA_TCGA-B2-4102 | C |
| TCGA_TCGA-B0-5083 | B |
| TCGA_TCGA-B0-4697 | A |
| TCGA_TCGA-B2-3924 | B |
| TCGA_TCGA-DV-A4VZ | C |
| TCGA_TCGA-B0-4821 | A |
| TCGA_TCGA-B0-4813 | B |
| TCGA_TCGA-CZ-5459 | B |
| TCGA_TCGA-B0-5108 | C |
| TCGA_TCGA-CZ-4862 | C |
| TCGA_TCGA-MM-A564 | B |
| TCGA_TCGA-CJ-4881 | A |
| TCGA_TCGA-B0-5077 | C |
| TCGA_TCGA-B0-5092 | B |
| TCGA_TCGA-EU-5905 | C |
| TCGA_TCGA-BP-5168 | C |
| TCGA_TCGA-B4-5832 | A |
| TCGA_TCGA-B8-5163 | A |
| TCGA_TCGA-DV-5575 | C |
| TCGA_TCGA-CW-5580 | C |
| TCGA_TCGA-B0-5692 | B |
| TCGA_TCGA-CJ-4640 | C |
| TCGA_TCGA-A3-3316 | A |
| TCGA_TCGA-BP-4797 | C |
| TCGA_TCGA-BP-5195 | C |
| TCGA_TCGA-B0-5691 | C |
| TCGA_TCGA-BP-5006 | B |
| TCGA_TCGA-CZ-5469 | A |
| TCGA_TCGA-BP-4999 | C |
| TCGA_TCGA-EU-5904 | C |
| TCGA_TCGA-BP-5189 | C |
| TCGA_TCGA-B0-5812 | C |
| TCGA_TCGA-A3-3385 | B |
| TCGA_TCGA-BP-4787 | A |
| TCGA_TCGA-BP-4325 | C |
| TCGA_TCGA-CJ-5677 | A |
| TCGA_TCGA-BP-4167 | A |
| TCGA_TCGA-CW-6097 | C |
| TCGA_TCGA-B8-5162 | B |
| TCGA_TCGA-CJ-6031 | B |
| TCGA_TCGA-CJ-4639 | C |
| TCGA_TCGA-BP-4759 | B |
| TCGA_TCGA-B8-4621 | B |
| TCGA_TCGA-CZ-5452 | B |
| TCGA_TCGA-B0-5699 | C |
| TCGA_TCGA-CZ-5460 | B |
| TCGA_TCGA-B0-4845 | B |
| TCGA_TCGA-MM-A563 | B |
| TCGA_TCGA-BP-4971 | B |
| TCGA_TCGA-B0-5712 | B |
| TCGA_TCGA-B0-4706 | B |
| TCGA_TCGA-CZ-5461 | C |
| TCGA_TCGA-6D-AA2E | B |
| TCGA_TCGA-EU-5907 | A |
| TCGA_TCGA-A3-3323 | C |
| TCGA_TCGA-CJ-4895 | A |
| TCGA_TCGA-CW-5591 | C |
| TCGA_TCGA-BP-4799 | A |
| TCGA_TCGA-B0-4847 | B |
| TCGA_TCGA-CW-6087 | C |
| TCGA_TCGA-BP-4173 | C |
| TCGA_TCGA-B8-4619 | A |
| TCGA_TCGA-BP-4176 | A |
| TCGA_TCGA-BP-4768 | C |
| TCGA_TCGA-B8-A54I | A |
| TCGA_TCGA-BP-5192 | C |
| TCGA_TCGA-BP-5181 | C |
| TCGA_TCGA-EU-5906 | C |
| TCGA_TCGA-B8-4620 | C |
| TCGA_TCGA-BP-4959 | C |
| TCGA_TCGA-CJ-4905 | C |
| TCGA_TCGA-CJ-6032 | C |
| TCGA_TCGA-B0-5703 | B |
| TCGA_TCGA-B8-A54F | A |
| TCGA_TCGA-B0-5095 | C |
| TCGA_TCGA-CZ-4861 | A |
| TCGA_TCGA-BP-4964 | C |
| TCGA_TCGA-B0-5402 | C |
| TCGA_TCGA-CJ-4891 | A |
| TCGA_TCGA-B0-4690 | A |
| TCGA_TCGA-A3-3374 | B |
| TCGA_TCGA-CZ-5470 | B |
| TCGA_TCGA-CZ-5463 | C |
| TCGA_TCGA-B8-4153 | B |
| TCGA_TCGA-B0-4698 | B |
| TCGA_TCGA-B0-5106 | A |
| TCGA_TCGA-CJ-4892 | C |
| TCGA_TCGA-CZ-4863 | C |
| TCGA_TCGA-BP-4330 | C |
| TCGA_TCGA-B0-4824 | B |
| TCGA_TCGA-CJ-4871 | C |
| TCGA_TCGA-B0-5690 | C |
| TCGA_TCGA-B8-5549 | C |
| TCGA_TCGA-DV-A4W0 | C |
| TCGA_TCGA-CZ-5989 | C |
| TCGA_TCGA-B0-5081 | B |
| TCGA_TCGA-CZ-4866 | C |
| TCGA_TCGA-BP-5182 | C |
| TCGA_TCGA-CJ-6033 | A |
| TCGA_TCGA-BP-4327 | B |
| TCGA_TCGA-BP-5200 | A |
| TCGA_TCGA-BP-4160 | C |
| TCGA_TCGA-B0-5120 | C |
| TCGA_TCGA-B0-5100 | C |
| TCGA_TCGA-B0-4945 | C |
| TCGA_TCGA-BP-4781 | C |
| TCGA_TCGA-CJ-5684 | C |
| TCGA_TCGA-BP-4346 | B |
| TCGA_TCGA-AK-3426 | B |
| TCGA_TCGA-CJ-5682 | C |
| TCGA_TCGA-A3-A6NL | C |
| TCGA_TCGA-CZ-5987 | B |
| TCGA_TCGA-A3-3382 | A |
| TCGA_TCGA-CJ-4636 | A |
| TCGA_TCGA-CW-5583 | C |
| TCGA_TCGA-B0-4849 | C |
| TCGA_TCGA-B0-4852 | B |
| TCGA_TCGA-BP-4995 | C |
| TCGA_TCGA-BP-4798 | B |
| TCGA_TCGA-B0-5700 | B |
| TCGA_TCGA-AK-3456 | B |
| TCGA_TCGA-CJ-4641 | A |
| TCGA_TCGA-BP-4335 | B |
| TCGA_TCGA-B8-4146 | C |
| TCGA_TCGA-B0-4694 | A |
| TCGA_TCGA-B0-4836 | A |
| TCGA_TCGA-CJ-4635 | C |
| TCGA_TCGA-BP-4975 | C |
| TCGA_TCGA-B8-A54E | B |
| TCGA_TCGA-A3-3376 | C |
| TCGA_TCGA-BP-4758 | B |
| TCGA_TCGA-B0-5121 | B |
| TCGA_TCGA-BP-5180 | B |
| TCGA_TCGA-DV-5566 | C |
| TCGA_TCGA-BP-4784 | C |
| TCGA_TCGA-A3-3331 | C |
| TCGA_TCGA-CZ-5455 | C |
| TCGA_TCGA-A3-3383 | B |
| TCGA_TCGA-CJ-4875 | B |
| TCGA_TCGA-B0-4811 | B |
| TCGA_TCGA-BP-4341 | B |
| TCGA_TCGA-B0-5099 | C |
| TCGA_TCGA-B0-4839 | B |
| TCGA_TCGA-CJ-5678 | B |
| TCGA_TCGA-DV-A4VX | B |
| TCGA_TCGA-CJ-6030 | A |
| TCGA_TCGA-B0-4818 | B |
| TCGA_TCGA-B0-5706 | B |
| TCGA_TCGA-BP-4973 | B |
| TCGA_TCGA-B8-5165 | C |
| TCGA_TCGA-CJ-4901 | B |
| TCGA_TCGA-B0-5110 | C |
| TCGA_TCGA-B0-4838 | C |
| TCGA_TCGA-BP-4968 | B |
| TCGA_TCGA-A3-A8OX | C |
| TCGA_TCGA-A3-A6NN | C |
| TCGA_TCGA-B0-4814 | A |
| TCGA_TCGA-B0-4823 | A |
| TCGA_TCGA-B0-5098 | C |
| TCGA_TCGA-B0-5711 | C |
| TCGA_TCGA-A3-3322 | B |
| TCGA_TCGA-B8-4151 | B |
| TCGA_TCGA-BP-4982 | C |
| TCGA_TCGA-B0-4713 | A |
| TCGA_TCGA-CJ-4902 | C |
| TCGA_TCGA-B0-5399 | B |
| TCGA_TCGA-B0-4707 | A |
| TCGA_TCGA-CZ-4865 | C |
| TCGA_TCGA-BP-4344 | C |
| TCGA_TCGA-DV-5565 | B |
| TCGA_TCGA-AK-3436 | A |
| TCGA_TCGA-A3-A6NJ | C |
| TCGA_TCGA-BP-5191 | A |
| TCGA_TCGA-B8-4154 | B |
| TCGA_TCGA-B0-4688 | C |
| TCGA_TCGA-BP-4986 | A |
| TCGA_TCGA-B0-5075 | B |
| TCGA_TCGA-BP-4775 | C |
| TCGA_TCGA-CJ-4888 | A |
| TCGA_TCGA-AK-3431 | A |
| TCGA_TCGA-BP-4338 | A |
| TCGA_TCGA-B0-4701 | B |
| TCGA_TCGA-A3-3325 | C |
| TCGA_TCGA-CW-6090 | A |
| TCGA_TCGA-BP-4177 | C |
| TCGA_TCGA-BP-4170 | C |
| TCGA_TCGA-A3-3357 | C |
| TCGA_TCGA-BP-4960 | B |
| TCGA_TCGA-AK-3440 | A |
| TCGA_TCGA-A3-3365 | C |
| TCGA_TCGA-DV-5569 | C |
| TCGA_TCGA-BP-5170 | C |
| TCGA_TCGA-CJ-6027 | A |
| TCGA_TCGA-BP-4983 | A |
| TCGA_TCGA-B4-5378 | C |
| TCGA_TCGA-A3-3317 | A |
| TCGA_TCGA-BP-4771 | B |
| TCGA_TCGA-A3-3352 | C |
| TCGA_TCGA-B0-5084 | B |
| TCGA_TCGA-BP-4159 | B |
| TCGA_TCGA-B8-A54J | C |
| TCGA_TCGA-CJ-4644 | C |
| TCGA_TCGA-B4-5836 | C |
| TCGA_TCGA-B0-5117 | B |
| TCGA_TCGA-CJ-5680 | C |
| TCGA_TCGA-CW-5589 | C |
| TCGA_TCGA-BP-5000 | B |
| TCGA_TCGA-A3-3346 | A |
| TCGA_TCGA-CJ-5689 | A |
| TCGA_TCGA-3Z-A93Z | C |
| TCGA_TCGA-BP-4807 | C |
| TCGA_TCGA-CJ-4908 | C |
| TCGA_TCGA-BP-4352 | A |
| TCGA_TCGA-A3-A8OU | C |
| TCGA_TCGA-AK-3434 | A |
| TCGA_TCGA-CJ-5683 | A |
| TCGA_TCGA-A3-3326 | B |
| TCGA_TCGA-CJ-5671 | A |
| TCGA_TCGA-AK-3425 | A |
| TCGA_TCGA-CZ-4858 | A |
| TCGA_TCGA-B0-4841 | B |
| TCGA_TCGA-CJ-4897 | C |
| TCGA_TCGA-BP-4776 | B |
| TCGA_TCGA-B4-5377 | C |
| TCGA_TCGA-CZ-5468 | A |
| TCGA_TCGA-CJ-4904 | C |
| TCGA_TCGA-BP-4756 | B |
| TCGA_TCGA-B0-5088 | B |
| TCGA_TCGA-B0-4837 | A |
| TCGA_TCGA-BP-5174 | B |
| TCGA_TCGA-BP-4803 | C |
| TCGA_TCGA-B0-5097 | A |
| TCGA_TCGA-B0-5119 | C |
| TCGA_TCGA-BP-4345 | C |
| TCGA_TCGA-AK-3453 | B |
| TCGA_TCGA-BP-4342 | A |
| TCGA_TCGA-B0-4844 | A |
| TCGA_TCGA-BP-4804 | B |
| TCGA_TCGA-CW-6088 | C |
| TCGA_TCGA-B0-5701 | A |
| TCGA_TCGA-CJ-5681 | A |
| TCGA_TCGA-A3-3319 | A |
| TCGA_TCGA-BP-5007 | C |
| TCGA_TCGA-B8-A54D | B |
| TCGA_TCGA-B0-4833 | B |
| TCGA_TCGA-BP-4347 | B |
| TCGA_TCGA-CW-5590 | A |
| TCGA_TCGA-B0-4822 | A |
| TCGA_TCGA-B0-4703 | A |
| TCGA_TCGA-BP-4770 | C |
| TCGA_TCGA-CJ-4637 | A |
| TCGA_TCGA-CJ-4885 | A |
| TCGA_TCGA-B8-5546 | A |
| TCGA_TCGA-B8-5545 | C |
| TCGA_TCGA-CJ-5676 | A |
| TCGA_TCGA-B0-5707 | B |
| TCGA_TCGA-BP-5190 | B |
| TCGA_TCGA-B0-5709 | A |
| TCGA_TCGA-B0-5109 | A |
| TCGA_TCGA-B4-5843 | C |
| TCGA_TCGA-B2-4098 | B |
| TCGA_TCGA-CJ-4893 | C |
| TCGA_TCGA-BP-4985 | A |
| TCGA_TCGA-BP-5001 | B |
| TCGA_TCGA-A3-3313 | B |
| TCGA_TCGA-BP-4169 | C |
| TCGA_TCGA-B8-5553 | C |
| TCGA_TCGA-AK-3443 | B |
| TCGA_TCGA-B0-4696 | C |
| TCGA_TCGA-DV-5576 | C |
| TCGA_TCGA-BP-4963 | C |
| TCGA_TCGA-BP-4354 | A |
| TCGA_TCGA-BP-4789 | C |
| TCGA_TCGA-CZ-5462 | A |
| TCGA_TCGA-B0-5705 | C |
| TCGA_TCGA-B0-4710 | C |
| TCGA_TCGA-BP-5004 | C |
| TCGA_TCGA-A3-3387 | C |
| TCGA_TCGA-BP-5175 | B |
| TCGA_TCGA-BP-4331 | C |
| TCGA_TCGA-BP-4174 | C |
| TCGA_TCGA-CJ-6028 | A |
| TCGA_TCGA-A3-3367 | C |
| TCGA_TCGA-A3-3351 | C |
| TCGA_TCGA-CJ-4890 | A |
| TCGA_TCGA-CW-5581 | C |
| TCGA_TCGA-B0-4815 | A |
| TCGA_TCGA-AK-3454 | A |
| TCGA_TCGA-BP-4782 | C |
| TCGA_TCGA-CZ-5451 | C |
| TCGA_TCGA-CJ-4878 | C |
| TCGA_TCGA-CJ-5672 | B |
| TCGA_TCGA-BP-4765 | C |
| TCGA_TCGA-BP-4970 | C |
| TCGA_TCGA-B0-4819 | B |
| TCGA_TCGA-G6-A5PC | B |
| TCGA_TCGA-BP-4992 | A |
| TCGA_TCGA-CZ-4864 | C |
| TCGA_TCGA-BP-5198 | C |
| TCGA_TCGA-BP-4349 | B |
| TCGA_TCGA-BP-4965 | C |
| TCGA_TCGA-B4-5835 | B |
| TCGA_TCGA-B0-5094 | A |
| TCGA_TCGA-DV-5567 | C |
| TCGA_TCGA-BP-4334 | B |
| TCGA_TCGA-BP-5187 | A |
| TCGA_TCGA-CZ-5988 | C |
| TCGA_TCGA-CJ-4894 | C |
| TCGA_TCGA-BP-4763 | C |
| TCGA_TCGA-B0-4828 | B |
| TCGA_TCGA-CJ-5679 | A |
| TCGA_TCGA-CJ-4869 | A |
| TCGA_TCGA-A3-3363 | B |
| TCGA_TCGA-BP-4777 | A |
| TCGA_TCGA-B0-4843 | A |
| TCGA_TCGA-BP-4766 | C |
| TCGA_TCGA-B8-A54H | C |
| TCGA_TCGA-BP-4774 | B |
| TCGA_TCGA-A3-3373 | C |
| TCGA_TCGA-CZ-4860 | A |
| TCGA_TCGA-AK-3451 | B |
| TCGA_TCGA-B0-5104 | B |
| TCGA_TCGA-BP-4351 | A |
| TCGA_TCGA-B2-4099 | B |
| TCGA_TCGA-B0-5694 | B |
| TCGA_TCGA-CJ-4903 | A |
| TCGA_TCGA-CZ-5457 | C |
| TCGA_TCGA-BP-4158 | B |
| TCGA_TCGA-A3-3320 | C |
| TCGA_TCGA-DV-5568 | A |
| TCGA_TCGA-CZ-5985 | C |
| TCGA_TCGA-BP-4166 | B |
| TCGA_TCGA-AS-3777 | A |
| TCGA_TCGA-CZ-5456 | A |
| TCGA_TCGA-BP-5183 | C |
| TCGA_TCGA-CZ-4854 | A |
| TCGA_TCGA-A3-3306 | C |
| TCGA_TCGA-B8-5158 | A |
| TCGA_TCGA-B0-5695 | C |
| TCGA_TCGA-BP-5176 | B |
| TCGA_TCGA-CZ-4859 | C |
| TCGA_TCGA-CW-5587 | C |
| TCGA_TCGA-B4-5844 | C |
| TCGA_TCGA-BP-4801 | C |
| TCGA_TCGA-A3-3307 | C |
| TCGA_TCGA-B4-5834 | C |
| TCGA_TCGA-G6-A8L6 | B |
| TCGA_TCGA-BP-4991 | C |
| TCGA_TCGA-A3-3347 | A |
| TCGA_TCGA-B2-5636 | C |
| TCGA_TCGA-A3-3308 | C |
| TCGA_TCGA-MM-A84U | B |
| TCGA_TCGA-CJ-4907 | C |
| TCGA_TCGA-BP-5196 | A |
| TCGA_TCGA-CJ-4889 | C |
| TCGA_TCGA-AK-3428 | C |
| TCGA_TCGA-CJ-4874 | C |
| TCGA_TCGA-A3-3328 | B |
| TCGA_TCGA-CJ-4872 | A |
| TCGA_TCGA-B8-A54K | C |
| TCGA_TCGA-CJ-4873 | B |
| TCGA_TCGA-G6-A8L7 | A |
| TCGA_TCGA-CZ-5467 | C |
| TCGA_TCGA-B8-5159 | C |
| TCGA_TCGA-A3-3358 | B |
| TCGA_TCGA-A3-3372 | C |
| TCGA_TCGA-BP-4974 | C |
| TCGA_TCGA-CW-5584 | C |
| TCGA_TCGA-B4-5838 | C |
| TCGA_TCGA-B0-4699 | C |
| TCGA_TCGA-B8-5550 | A |
| TCGA_TCGA-A3-A8OW | C |
| TCGA_TCGA-BP-4967 | C |
| TCGA_TCGA-BP-5201 | B |
| TCGA_TCGA-T7-A92I | B |
| TCGA_TCGA-B0-5085 | B |
| TCGA_TCGA-CZ-4853 | B |
| TCGA_TCGA-A3-A6NI | C |
| TCGA_TCGA-AK-3465 | B |
| TCGA_TCGA-BP-4989 | B |
| TCGA_TCGA-G6-A8L8 | B |
| TCGA_TCGA-BP-4162 | C |
| TCGA_TCGA-BP-5173 | C |
| TCGA_TCGA-B8-A8YJ | A |
| TCGA_TCGA-B0-5080 | A |
| TCGA_TCGA-BP-4976 | C |
| TCGA_TCGA-BP-4163 | C |
| TCGA_TCGA-CJ-4899 | C |
| TCGA_TCGA-BP-4977 | B |
| TCGA_TCGA-A3-3335 | B |
| TCGA_TCGA-CJ-4634 | C |
| TCGA_TCGA-B2-4101 | A |
| TCGA_TCGA-BP-5185 | B |
| TCGA_TCGA-AK-3427 | A |
| TCGA_TCGA-BP-4998 | C |
| TCGA_TCGA-BP-5202 | C |
| TCGA_TCGA-B8-5164 | C |
| TCGA_TCGA-DV-5573 | A |
| TCGA_TCGA-B0-5697 | C |
| TCGA_TCGA-BP-4993 | C |
| TCGA_TCGA-B0-4817 | B |
| TCGA_TCGA-BP-4355 | B |
| TCGA_TCGA-A3-3359 | C |
| TCGA_TCGA-AK-3433 | B |
| TCGA_TCGA-B0-4827 | C |
| TCGA_TCGA-B2-A4SR | C |
| TCGA_TCGA-B2-5641 | C |
| TCGA_TCGA-CJ-4882 | A |
| TCGA_TCGA-BP-4795 | C |
| TCGA_TCGA-CW-5588 | C |
| TCGA_TCGA-CZ-5464 | B |
| TCGA_TCGA-BP-4762 | C |
| TCGA_TCGA-BP-5178 | A |
| TCGA_TCGA-B0-5115 | C |
| TCGA_TCGA-B8-4622 | B |
| TCGA_TCGA-B0-5102 | C |
| TCGA_TCGA-BP-5008 | C |
| TCGA_TCGA-CJ-4642 | B |
| TCGA_TCGA-A3-3362 | C |
| TCGA_TCGA-B2-5639 | C |
| TCGA_TCGA-CZ-5458 | C |
| TCGA_TCGA-AS-3778 | C |
| TCGA_TCGA-B0-4693 | C |
| TCGA_TCGA-CJ-4884 | B |
| TCGA_TCGA-B0-5710 | C |
| TCGA_TCGA-BP-4353 | A |
| TCGA_TCGA-GK-A6C7 | A |
| TCGA_TCGA-BP-4761 | A |
| TCGA_TCGA-A3-3329 | B |
| TCGA_TCGA-BP-4332 | C |
| TCGA_TCGA-CJ-4643 | C |
| TCGA_TCGA-B8-A54G | B |
| TCGA_TCGA-BP-4961 | C |
| TCGA_TCGA-B0-5698 | B |
| TCGA_TCGA-BP-5199 | C |
| TCGA_TCGA-CW-6093 | C |
| TCGA_TCGA-B0-4700 | A |
| TCGA_TCGA-AK-3460 | C |
| TCGA_TCGA-AK-3458 | B |
| TCGA_TCGA-CZ-5984 | A |
| TCGA_TCGA-A3-3324 | C |
| TCGA_TCGA-CJ-4912 | B |
| TCGA_TCGA-CZ-5453 | B |
| TCGA_TCGA-A3-3378 | C |
| TCGA_TCGA-A3-3343 | B |
| TCGA_TCGA-CJ-4887 | B |
| TCGA_TCGA-DV-5574 | A |
| TCGA_TCGA-A3-A8CQ | C |
| TCGA_TCGA-B0-4816 | B |
| TCGA_TCGA-A3-3311 | C |
| TCGA_TCGA-CJ-4868 | A |
| TCGA_TCGA-B8-4148 | C |
| TCGA_TCGA-CJ-4886 | C |
| TCGA_TCGA-AK-3429 | B |
| TCGA_TCGA-CZ-5465 | C |
| TCGA_TCGA-B0-4714 | B |
| TCGA_TCGA-AK-3461 | C |
| TCGA_TCGA-CZ-5454 | B |
| TCGA_TCGA-BP-4790 | C |
| TCGA_TCGA-B0-5107 | B |
| GSE29609_GSM733579 | B |
| GSE29609_GSM733580 | B |
| GSE29609_GSM733581 | A |
| GSE29609_GSM733582 | C |
| GSE29609_GSM733583 | A |
| GSE29609_GSM733584 | C |
| GSE29609_GSM733585 | A |
| GSE29609_GSM733586 | A |
| GSE29609_GSM733587 | A |
| GSE29609_GSM733588 | A |
| GSE29609_GSM733589 | B |
| GSE29609_GSM733590 | A |
| GSE29609_GSM733591 | B |
| GSE29609_GSM733592 | A |
| GSE29609_GSM733593 | A |
| GSE29609_GSM733594 | A |
| GSE29609_GSM733595 | A |
| GSE29609_GSM733596 | A |
| GSE29609_GSM733597 | B |
| GSE29609_GSM733598 | A |
| GSE29609_GSM733599 | A |
| GSE29609_GSM733600 | C |
| GSE29609_GSM733601 | A |
| GSE29609_GSM733602 | A |
| GSE29609_GSM733603 | B |
| GSE29609_GSM733604 | C |
| GSE29609_GSM733605 | C |
| GSE29609_GSM733606 | A |
| GSE29609_GSM733607 | A |
| GSE29609_GSM733608 | A |
| GSE29609_GSM733609 | A |
| GSE29609_GSM733610 | A |
| GSE29609_GSM733611 | B |
| GSE29609_GSM733612 | C |
| GSE29609_GSM733613 | B |
| GSE29609_GSM733614 | A |
| GSE29609_GSM733615 | B |
| GSE29609_GSM733616 | A |
| GSE29609_GSM733617 | A |

**Table S5.** Identification of m6A-related gene clusters.

| **ID** | **geneCluster** |
| --- | --- |
| TCGA_TCGA-CJ-4920 | A |
| TCGA_TCGA-BP-5177 | B |
| TCGA_TCGA-B0-4842 | C |
| TCGA_TCGA-BP-4343 | A |
| TCGA_TCGA-MW-A4EC | B |
| TCGA_TCGA-B8-4143 | B |
| TCGA_TCGA-CJ-5675 | B |
| TCGA_TCGA-BP-4760 | A |
| TCGA_TCGA-B0-5113 | A |
| TCGA_TCGA-BP-4340 | A |
| TCGA_TCGA-B8-A7U6 | A |
| TCGA_TCGA-B8-5551 | B |
| TCGA_TCGA-B0-5400 | C |
| TCGA_TCGA-BP-4994 | A |
| TCGA_TCGA-A3-3349 | A |
| TCGA_TCGA-AK-3455 | B |
| TCGA_TCGA-CW-5585 | A |
| TCGA_TCGA-BP-4981 | C |
| TCGA_TCGA-BP-4337 | C |
| TCGA_TCGA-CJ-5686 | A |
| TCGA_TCGA-A3-A8OV | B |
| TCGA_TCGA-AK-3447 | C |
| TCGA_TCGA-B0-5702 | C |
| TCGA_TCGA-CJ-4918 | B |
| TCGA_TCGA-B0-4848 | A |
| TCGA_TCGA-BP-5194 | A |
| TCGA_TCGA-B2-5635 | A |
| TCGA_TCGA-B0-5096 | B |
| TCGA_TCGA-CZ-5986 | A |
| TCGA_TCGA-A3-3380 | B |
| TCGA_TCGA-BP-4329 | B |
| TCGA_TCGA-B0-4712 | B |
| TCGA_TCGA-B0-4691 | C |
| TCGA_TCGA-CZ-5982 | A |
| TCGA_TCGA-BP-4962 | B |
| TCGA_TCGA-CZ-5466 | B |
| TCGA_TCGA-AK-3450 | A |
| TCGA_TCGA-CJ-4876 | B |
| TCGA_TCGA-B0-4834 | C |
| TCGA_TCGA-B0-5713 | A |
| TCGA_TCGA-BP-5009 | C |
| TCGA_TCGA-CJ-4900 | C |
| TCGA_TCGA-BP-5186 | A |
| TCGA_TCGA-A3-3370 | A |
| TCGA_TCGA-B0-4810 | B |
| TCGA_TCGA-CJ-4916 | B |
| TCGA_TCGA-B0-4846 | A |
| TCGA_TCGA-CJ-4638 | C |
| TCGA_TCGA-BP-5184 | A |
| TCGA_TCGA-B0-5116 | B |
| TCGA_TCGA-CZ-4857 | B |
| TCGA_TCGA-B0-4718 | C |
| TCGA_TCGA-B0-5696 | B |
| TCGA_TCGA-BP-4326 | B |
| TCGA_TCGA-CZ-4856 | A |
| TCGA_TCGA-BP-4972 | A |
| TCGA_TCGA-BP-5169 | C |
| TCGA_TCGA-B2-5633 | A |
| TCGA_TCGA-B0-5693 | A |
| TCGA_TCGA-BP-4969 | C |
| TCGA_TCGA-BP-4165 | C |
| TCGA_TCGA-CJ-4870 | B |
| TCGA_TCGA-B8-5552 | A |
| TCGA_TCGA-BP-4164 | A |
| TCGA_TCGA-BP-4987 | A |
| TCGA_TCGA-BP-4769 | A |
| TCGA_TCGA-BP-4161 | A |
| TCGA_TCGA-BP-5010 | C |
| TCGA_TCGA-B2-3923 | B |
| TCGA_TCGA-AK-3445 | B |
| TCGA_TCGA-B2-4102 | A |
| TCGA_TCGA-B0-5083 | C |
| TCGA_TCGA-B0-4697 | C |
| TCGA_TCGA-B2-3924 | A |
| TCGA_TCGA-DV-A4VZ | B |
| TCGA_TCGA-B0-4821 | C |
| TCGA_TCGA-B0-4813 | C |
| TCGA_TCGA-CZ-5459 | B |
| TCGA_TCGA-B0-5108 | A |
| TCGA_TCGA-CZ-4862 | A |
| TCGA_TCGA-MM-A564 | C |
| TCGA_TCGA-CJ-4881 | B |
| TCGA_TCGA-B0-5077 | B |
| TCGA_TCGA-B0-5092 | B |
| TCGA_TCGA-EU-5905 | A |
| TCGA_TCGA-BP-5168 | B |
| TCGA_TCGA-B4-5832 | A |
| TCGA_TCGA-B8-5163 | A |
| TCGA_TCGA-DV-5575 | A |
| TCGA_TCGA-CW-5580 | A |
| TCGA_TCGA-B0-5692 | B |
| TCGA_TCGA-CJ-4640 | A |
| TCGA_TCGA-A3-3316 | B |
| TCGA_TCGA-BP-4797 | B |
| TCGA_TCGA-BP-5195 | A |
| TCGA_TCGA-B0-5691 | A |
| TCGA_TCGA-BP-5006 | B |
| TCGA_TCGA-CZ-5469 | C |
| TCGA_TCGA-BP-4999 | A |
| TCGA_TCGA-EU-5904 | A |
| TCGA_TCGA-BP-5189 | A |
| TCGA_TCGA-B0-5812 | A |
| TCGA_TCGA-A3-3385 | B |
| TCGA_TCGA-BP-4787 | B |
| TCGA_TCGA-BP-4325 | A |
| TCGA_TCGA-CJ-5677 | B |
| TCGA_TCGA-BP-4167 | C |
| TCGA_TCGA-CW-6097 | A |
| TCGA_TCGA-B8-5162 | B |
| TCGA_TCGA-CJ-6031 | B |
| TCGA_TCGA-CJ-4639 | A |
| TCGA_TCGA-BP-4759 | C |
| TCGA_TCGA-B8-4621 | B |
| TCGA_TCGA-CZ-5452 | B |
| TCGA_TCGA-B0-5699 | A |
| TCGA_TCGA-CZ-5460 | B |
| TCGA_TCGA-B0-4845 | B |
| TCGA_TCGA-MM-A563 | B |
| TCGA_TCGA-BP-4971 | C |
| TCGA_TCGA-B0-5712 | B |
| TCGA_TCGA-B0-4706 | B |
| TCGA_TCGA-CZ-5461 | A |
| TCGA_TCGA-6D-AA2E | B |
| TCGA_TCGA-EU-5907 | A |
| TCGA_TCGA-A3-3323 | A |
| TCGA_TCGA-CJ-4895 | B |
| TCGA_TCGA-CW-5591 | A |
| TCGA_TCGA-BP-4799 | C |
| TCGA_TCGA-B0-4847 | C |
| TCGA_TCGA-CW-6087 | A |
| TCGA_TCGA-BP-4173 | A |
| TCGA_TCGA-B8-4619 | B |
| TCGA_TCGA-BP-4176 | A |
| TCGA_TCGA-BP-4768 | B |
| TCGA_TCGA-B8-A54I | C |
| TCGA_TCGA-BP-5192 | A |
| TCGA_TCGA-BP-5181 | A |
| TCGA_TCGA-EU-5906 | A |
| TCGA_TCGA-B8-4620 | B |
| TCGA_TCGA-BP-4959 | B |
| TCGA_TCGA-CJ-4905 | A |
| TCGA_TCGA-CJ-6032 | A |
| TCGA_TCGA-B0-5703 | B |
| TCGA_TCGA-B8-A54F | A |
| TCGA_TCGA-B0-5095 | A |
| TCGA_TCGA-CZ-4861 | B |
| TCGA_TCGA-BP-4964 | B |
| TCGA_TCGA-B0-5402 | A |
| TCGA_TCGA-CJ-4891 | C |
| TCGA_TCGA-B0-4690 | A |
| TCGA_TCGA-A3-3374 | C |
| TCGA_TCGA-CZ-5470 | B |
| TCGA_TCGA-CZ-5463 | A |
| TCGA_TCGA-B8-4153 | C |
| TCGA_TCGA-B0-4698 | B |
| TCGA_TCGA-B0-5106 | B |
| TCGA_TCGA-CJ-4892 | B |
| TCGA_TCGA-CZ-4863 | A |
| TCGA_TCGA-BP-4330 | A |
| TCGA_TCGA-B0-4824 | B |
| TCGA_TCGA-CJ-4871 | B |
| TCGA_TCGA-B0-5690 | A |
| TCGA_TCGA-B8-5549 | A |
| TCGA_TCGA-DV-A4W0 | A |
| TCGA_TCGA-CZ-5989 | A |
| TCGA_TCGA-B0-5081 | C |
| TCGA_TCGA-CZ-4866 | A |
| TCGA_TCGA-BP-5182 | A |
| TCGA_TCGA-CJ-6033 | B |
| TCGA_TCGA-BP-4327 | B |
| TCGA_TCGA-BP-5200 | A |
| TCGA_TCGA-BP-4160 | A |
| TCGA_TCGA-B0-5120 | A |
| TCGA_TCGA-B0-5100 | B |
| TCGA_TCGA-B0-4945 | A |
| TCGA_TCGA-BP-4781 | A |
| TCGA_TCGA-CJ-5684 | B |
| TCGA_TCGA-BP-4346 | B |
| TCGA_TCGA-AK-3426 | C |
| TCGA_TCGA-CJ-5682 | B |
| TCGA_TCGA-A3-A6NL | A |
| TCGA_TCGA-CZ-5987 | B |
| TCGA_TCGA-A3-3382 | A |
| TCGA_TCGA-CJ-4636 | B |
| TCGA_TCGA-CW-5583 | A |
| TCGA_TCGA-B0-4849 | B |
| TCGA_TCGA-B0-4852 | A |
| TCGA_TCGA-BP-4995 | A |
| TCGA_TCGA-BP-4798 | B |
| TCGA_TCGA-B0-5700 | B |
| TCGA_TCGA-AK-3456 | B |
| TCGA_TCGA-CJ-4641 | A |
| TCGA_TCGA-BP-4335 | C |
| TCGA_TCGA-B8-4146 | A |
| TCGA_TCGA-B0-4694 | B |
| TCGA_TCGA-B0-4836 | B |
| TCGA_TCGA-CJ-4635 | A |
| TCGA_TCGA-BP-4975 | A |
| TCGA_TCGA-B8-A54E | B |
| TCGA_TCGA-A3-3376 | A |
| TCGA_TCGA-BP-4758 | C |
| TCGA_TCGA-B0-5121 | C |
| TCGA_TCGA-BP-5180 | B |
| TCGA_TCGA-DV-5566 | B |
| TCGA_TCGA-BP-4784 | A |
| TCGA_TCGA-A3-3331 | A |
| TCGA_TCGA-CZ-5455 | A |
| TCGA_TCGA-A3-3383 | B |
| TCGA_TCGA-CJ-4875 | C |
| TCGA_TCGA-B0-4811 | C |
| TCGA_TCGA-BP-4341 | B |
| TCGA_TCGA-B0-5099 | B |
| TCGA_TCGA-B0-4839 | C |
| TCGA_TCGA-CJ-5678 | B |
| TCGA_TCGA-DV-A4VX | C |
| TCGA_TCGA-CJ-6030 | A |
| TCGA_TCGA-B0-4818 | B |
| TCGA_TCGA-B0-5706 | B |
| TCGA_TCGA-BP-4973 | A |
| TCGA_TCGA-B8-5165 | A |
| TCGA_TCGA-CJ-4901 | B |
| TCGA_TCGA-B0-5110 | A |
| TCGA_TCGA-B0-4838 | A |
| TCGA_TCGA-BP-4968 | B |
| TCGA_TCGA-A3-A8OX | A |
| TCGA_TCGA-A3-A6NN | A |
| TCGA_TCGA-B0-4814 | B |
| TCGA_TCGA-B0-4823 | A |
| TCGA_TCGA-B0-5098 | B |
| TCGA_TCGA-B0-5711 | A |
| TCGA_TCGA-A3-3322 | B |
| TCGA_TCGA-B8-4151 | B |
| TCGA_TCGA-BP-4982 | A |
| TCGA_TCGA-B0-4713 | C |
| TCGA_TCGA-CJ-4902 | B |
| TCGA_TCGA-B0-5399 | B |
| TCGA_TCGA-B0-4707 | C |
| TCGA_TCGA-CZ-4865 | A |
| TCGA_TCGA-BP-4344 | A |
| TCGA_TCGA-DV-5565 | B |
| TCGA_TCGA-AK-3436 | B |
| TCGA_TCGA-A3-A6NJ | A |
| TCGA_TCGA-BP-5191 | C |
| TCGA_TCGA-B8-4154 | B |
| TCGA_TCGA-B0-4688 | A |
| TCGA_TCGA-BP-4986 | A |
| TCGA_TCGA-B0-5075 | B |
| TCGA_TCGA-BP-4775 | A |
| TCGA_TCGA-CJ-4888 | B |
| TCGA_TCGA-AK-3431 | A |
| TCGA_TCGA-BP-4338 | B |
| TCGA_TCGA-B0-4701 | B |
| TCGA_TCGA-A3-3325 | B |
| TCGA_TCGA-CW-6090 | B |
| TCGA_TCGA-BP-4177 | A |
| TCGA_TCGA-BP-4170 | A |
| TCGA_TCGA-A3-3357 | B |
| TCGA_TCGA-BP-4960 | C |
| TCGA_TCGA-AK-3440 | C |
| TCGA_TCGA-A3-3365 | A |
| TCGA_TCGA-DV-5569 | A |
| TCGA_TCGA-BP-5170 | B |
| TCGA_TCGA-CJ-6027 | B |
| TCGA_TCGA-BP-4983 | B |
| TCGA_TCGA-B4-5378 | A |
| TCGA_TCGA-A3-3317 | A |
| TCGA_TCGA-BP-4771 | B |
| TCGA_TCGA-A3-3352 | A |
| TCGA_TCGA-B0-5084 | C |
| TCGA_TCGA-BP-4159 | C |
| TCGA_TCGA-B8-A54J | A |
| TCGA_TCGA-CJ-4644 | A |
| TCGA_TCGA-B4-5836 | A |
| TCGA_TCGA-B0-5117 | C |
| TCGA_TCGA-CJ-5680 | A |
| TCGA_TCGA-CW-5589 | A |
| TCGA_TCGA-BP-5000 | C |
| TCGA_TCGA-A3-3346 | B |
| TCGA_TCGA-CJ-5689 | B |
| TCGA_TCGA-3Z-A93Z | B |
| TCGA_TCGA-BP-4807 | A |
| TCGA_TCGA-CJ-4908 | A |
| TCGA_TCGA-BP-4352 | C |
| TCGA_TCGA-A3-A8OU | B |
| TCGA_TCGA-AK-3434 | B |
| TCGA_TCGA-CJ-5683 | B |
| TCGA_TCGA-A3-3326 | B |
| TCGA_TCGA-CJ-5671 | B |
| TCGA_TCGA-AK-3425 | B |
| TCGA_TCGA-CZ-4858 | B |
| TCGA_TCGA-B0-4841 | C |
| TCGA_TCGA-CJ-4897 | A |
| TCGA_TCGA-BP-4776 | C |
| TCGA_TCGA-B4-5377 | A |
| TCGA_TCGA-CZ-5468 | B |
| TCGA_TCGA-CJ-4904 | A |
| TCGA_TCGA-BP-4756 | B |
| TCGA_TCGA-B0-5088 | C |
| TCGA_TCGA-B0-4837 | B |
| TCGA_TCGA-BP-5174 | C |
| TCGA_TCGA-BP-4803 | B |
| TCGA_TCGA-B0-5097 | A |
| TCGA_TCGA-B0-5119 | A |
| TCGA_TCGA-BP-4345 | B |
| TCGA_TCGA-AK-3453 | C |
| TCGA_TCGA-BP-4342 | B |
| TCGA_TCGA-B0-4844 | B |
| TCGA_TCGA-BP-4804 | B |
| TCGA_TCGA-CW-6088 | A |
| TCGA_TCGA-B0-5701 | B |
| TCGA_TCGA-CJ-5681 | A |
| TCGA_TCGA-A3-3319 | B |
| TCGA_TCGA-BP-5007 | A |
| TCGA_TCGA-B8-A54D | B |
| TCGA_TCGA-B0-4833 | C |
| TCGA_TCGA-BP-4347 | A |
| TCGA_TCGA-CW-5590 | B |
| TCGA_TCGA-B0-4822 | C |
| TCGA_TCGA-B0-4703 | A |
| TCGA_TCGA-BP-4770 | B |
| TCGA_TCGA-CJ-4637 | B |
| TCGA_TCGA-CJ-4885 | B |
| TCGA_TCGA-B8-5546 | A |
| TCGA_TCGA-B8-5545 | A |
| TCGA_TCGA-CJ-5676 | C |
| TCGA_TCGA-B0-5707 | B |
| TCGA_TCGA-BP-5190 | C |
| TCGA_TCGA-B0-5709 | A |
| TCGA_TCGA-B0-5109 | B |
| TCGA_TCGA-B4-5843 | A |
| TCGA_TCGA-B2-4098 | C |
| TCGA_TCGA-CJ-4893 | A |
| TCGA_TCGA-BP-4985 | B |
| TCGA_TCGA-BP-5001 | C |
| TCGA_TCGA-A3-3313 | B |
| TCGA_TCGA-BP-4169 | A |
| TCGA_TCGA-B8-5553 | A |
| TCGA_TCGA-AK-3443 | B |
| TCGA_TCGA-B0-4696 | B |
| TCGA_TCGA-DV-5576 | A |
| TCGA_TCGA-BP-4963 | B |
| TCGA_TCGA-BP-4354 | A |
| TCGA_TCGA-BP-4789 | A |
| TCGA_TCGA-CZ-5462 | A |
| TCGA_TCGA-B0-5705 | A |
| TCGA_TCGA-B0-4710 | A |
| TCGA_TCGA-BP-5004 | A |
| TCGA_TCGA-A3-3387 | A |
| TCGA_TCGA-BP-5175 | C |
| TCGA_TCGA-BP-4331 | A |
| TCGA_TCGA-BP-4174 | A |
| TCGA_TCGA-CJ-6028 | A |
| TCGA_TCGA-A3-3367 | A |
| TCGA_TCGA-A3-3351 | A |
| TCGA_TCGA-CJ-4890 | B |
| TCGA_TCGA-CW-5581 | A |
| TCGA_TCGA-B0-4815 | B |
| TCGA_TCGA-AK-3454 | C |
| TCGA_TCGA-BP-4782 | A |
| TCGA_TCGA-CZ-5451 | A |
| TCGA_TCGA-CJ-4878 | B |
| TCGA_TCGA-CJ-5672 | B |
| TCGA_TCGA-BP-4765 | A |
| TCGA_TCGA-BP-4970 | A |
| TCGA_TCGA-B0-4819 | C |
| TCGA_TCGA-G6-A5PC | C |
| TCGA_TCGA-BP-4992 | C |
| TCGA_TCGA-CZ-4864 | A |
| TCGA_TCGA-BP-5198 | B |
| TCGA_TCGA-BP-4349 | B |
| TCGA_TCGA-BP-4965 | A |
| TCGA_TCGA-B4-5835 | B |
| TCGA_TCGA-B0-5094 | B |
| TCGA_TCGA-DV-5567 | A |
| TCGA_TCGA-BP-4334 | C |
| TCGA_TCGA-BP-5187 | A |
| TCGA_TCGA-CZ-5988 | A |
| TCGA_TCGA-CJ-4894 | A |
| TCGA_TCGA-BP-4763 | A |
| TCGA_TCGA-B0-4828 | B |
| TCGA_TCGA-CJ-5679 | C |
| TCGA_TCGA-CJ-4869 | B |
| TCGA_TCGA-A3-3363 | B |
| TCGA_TCGA-BP-4777 | B |
| TCGA_TCGA-B0-4843 | C |
| TCGA_TCGA-BP-4766 | A |
| TCGA_TCGA-B8-A54H | A |
| TCGA_TCGA-BP-4774 | B |
| TCGA_TCGA-A3-3373 | A |
| TCGA_TCGA-CZ-4860 | B |
| TCGA_TCGA-AK-3451 | C |
| TCGA_TCGA-B0-5104 | B |
| TCGA_TCGA-BP-4351 | B |
| TCGA_TCGA-B2-4099 | B |
| TCGA_TCGA-B0-5694 | B |
| TCGA_TCGA-CJ-4903 | B |
| TCGA_TCGA-CZ-5457 | A |
| TCGA_TCGA-BP-4158 | B |
| TCGA_TCGA-A3-3320 | A |
| TCGA_TCGA-DV-5568 | B |
| TCGA_TCGA-CZ-5985 | A |
| TCGA_TCGA-BP-4166 | B |
| TCGA_TCGA-AS-3777 | C |
| TCGA_TCGA-CZ-5456 | B |
| TCGA_TCGA-BP-5183 | B |
| TCGA_TCGA-CZ-4854 | B |
| TCGA_TCGA-A3-3306 | A |
| TCGA_TCGA-B8-5158 | A |
| TCGA_TCGA-B0-5695 | A |
| TCGA_TCGA-BP-5176 | B |
| TCGA_TCGA-CZ-4859 | A |
| TCGA_TCGA-CW-5587 | A |
| TCGA_TCGA-B4-5844 | A |
| TCGA_TCGA-BP-4801 | A |
| TCGA_TCGA-A3-3307 | A |
| TCGA_TCGA-B4-5834 | A |
| TCGA_TCGA-G6-A8L6 | B |
| TCGA_TCGA-BP-4991 | A |
| TCGA_TCGA-A3-3347 | A |
| TCGA_TCGA-B2-5636 | A |
| TCGA_TCGA-A3-3308 | A |
| TCGA_TCGA-MM-A84U | C |
| TCGA_TCGA-CJ-4907 | A |
| TCGA_TCGA-BP-5196 | B |
| TCGA_TCGA-CJ-4889 | A |
| TCGA_TCGA-AK-3428 | A |
| TCGA_TCGA-CJ-4874 | A |
| TCGA_TCGA-A3-3328 | B |
| TCGA_TCGA-CJ-4872 | B |
| TCGA_TCGA-B8-A54K | A |
| TCGA_TCGA-CJ-4873 | C |
| TCGA_TCGA-G6-A8L7 | C |
| TCGA_TCGA-CZ-5467 | A |
| TCGA_TCGA-B8-5159 | A |
| TCGA_TCGA-A3-3358 | B |
| TCGA_TCGA-A3-3372 | A |
| TCGA_TCGA-BP-4974 | A |
| TCGA_TCGA-CW-5584 | A |
| TCGA_TCGA-B4-5838 | A |
| TCGA_TCGA-B0-4699 | B |
| TCGA_TCGA-B8-5550 | A |
| TCGA_TCGA-A3-A8OW | A |
| TCGA_TCGA-BP-4967 | B |
| TCGA_TCGA-BP-5201 | B |
| TCGA_TCGA-T7-A92I | A |
| TCGA_TCGA-B0-5085 | C |
| TCGA_TCGA-CZ-4853 | B |
| TCGA_TCGA-A3-A6NI | B |
| TCGA_TCGA-AK-3465 | C |
| TCGA_TCGA-BP-4989 | C |
| TCGA_TCGA-G6-A8L8 | B |
| TCGA_TCGA-BP-4162 | A |
| TCGA_TCGA-BP-5173 | B |
| TCGA_TCGA-B8-A8YJ | A |
| TCGA_TCGA-B0-5080 | A |
| TCGA_TCGA-BP-4976 | A |
| TCGA_TCGA-BP-4163 | A |
| TCGA_TCGA-CJ-4899 | A |
| TCGA_TCGA-BP-4977 | B |
| TCGA_TCGA-A3-3335 | B |
| TCGA_TCGA-CJ-4634 | A |
| TCGA_TCGA-B2-4101 | A |
| TCGA_TCGA-BP-5185 | C |
| TCGA_TCGA-AK-3427 | C |
| TCGA_TCGA-BP-4998 | A |
| TCGA_TCGA-BP-5202 | A |
| TCGA_TCGA-B8-5164 | A |
| TCGA_TCGA-DV-5573 | A |
| TCGA_TCGA-B0-5697 | B |
| TCGA_TCGA-BP-4993 | B |
| TCGA_TCGA-B0-4817 | C |
| TCGA_TCGA-BP-4355 | B |
| TCGA_TCGA-A3-3359 | A |
| TCGA_TCGA-AK-3433 | C |
| TCGA_TCGA-B0-4827 | B |
| TCGA_TCGA-B2-A4SR | A |
| TCGA_TCGA-B2-5641 | A |
| TCGA_TCGA-CJ-4882 | B |
| TCGA_TCGA-BP-4795 | A |
| TCGA_TCGA-CW-5588 | A |
| TCGA_TCGA-CZ-5464 | B |
| TCGA_TCGA-BP-4762 | A |
| TCGA_TCGA-BP-5178 | C |
| TCGA_TCGA-B0-5115 | A |
| TCGA_TCGA-B8-4622 | A |
| TCGA_TCGA-B0-5102 | B |
| TCGA_TCGA-BP-5008 | A |
| TCGA_TCGA-CJ-4642 | C |
| TCGA_TCGA-A3-3362 | A |
| TCGA_TCGA-B2-5639 | B |
| TCGA_TCGA-CZ-5458 | A |
| TCGA_TCGA-AS-3778 | A |
| TCGA_TCGA-B0-4693 | A |
| TCGA_TCGA-CJ-4884 | B |
| TCGA_TCGA-B0-5710 | A |
| TCGA_TCGA-BP-4353 | A |
| TCGA_TCGA-GK-A6C7 | A |
| TCGA_TCGA-BP-4761 | C |
| TCGA_TCGA-A3-3329 | B |
| TCGA_TCGA-BP-4332 | A |
| TCGA_TCGA-CJ-4643 | A |
| TCGA_TCGA-B8-A54G | B |
| TCGA_TCGA-BP-4961 | A |
| TCGA_TCGA-B0-5698 | B |
| TCGA_TCGA-BP-5199 | B |
| TCGA_TCGA-CW-6093 | A |
| TCGA_TCGA-B0-4700 | B |
| TCGA_TCGA-AK-3460 | B |
| TCGA_TCGA-AK-3458 | B |
| TCGA_TCGA-CZ-5984 | A |
| TCGA_TCGA-A3-3324 | B |
| TCGA_TCGA-CJ-4912 | C |
| TCGA_TCGA-CZ-5453 | B |
| TCGA_TCGA-A3-3378 | A |
| TCGA_TCGA-A3-3343 | A |
| TCGA_TCGA-CJ-4887 | C |
| TCGA_TCGA-DV-5574 | A |
| TCGA_TCGA-A3-A8CQ | A |
| TCGA_TCGA-B0-4816 | B |
| TCGA_TCGA-A3-3311 | A |
| TCGA_TCGA-CJ-4868 | B |
| TCGA_TCGA-B8-4148 | A |
| TCGA_TCGA-CJ-4886 | A |
| TCGA_TCGA-AK-3429 | B |
| TCGA_TCGA-CZ-5465 | A |
| TCGA_TCGA-B0-4714 | C |
| TCGA_TCGA-AK-3461 | A |
| TCGA_TCGA-CZ-5454 | B |
| TCGA_TCGA-BP-4790 | A |
| TCGA_TCGA-B0-5107 | C |
| GSE29609_GSM733579 | B |
| GSE29609_GSM733580 | B |
| GSE29609_GSM733581 | B |
| GSE29609_GSM733582 | B |
| GSE29609_GSM733583 | B |
| GSE29609_GSM733584 | B |
| GSE29609_GSM733585 | B |
| GSE29609_GSM733586 | B |
| GSE29609_GSM733587 | B |
| GSE29609_GSM733588 | B |
| GSE29609_GSM733589 | B |
| GSE29609_GSM733590 | B |
| GSE29609_GSM733591 | B |
| GSE29609_GSM733592 | A |
| GSE29609_GSM733593 | B |
| GSE29609_GSM733594 | B |
| GSE29609_GSM733595 | B |
| GSE29609_GSM733596 | B |
| GSE29609_GSM733597 | B |
| GSE29609_GSM733598 | B |
| GSE29609_GSM733599 | B |
| GSE29609_GSM733600 | A |
| GSE29609_GSM733601 | B |
| GSE29609_GSM733602 | B |
| GSE29609_GSM733603 | B |
| GSE29609_GSM733604 | B |
| GSE29609_GSM733605 | A |
| GSE29609_GSM733606 | B |
| GSE29609_GSM733607 | B |
| GSE29609_GSM733608 | B |
| GSE29609_GSM733609 | B |
| GSE29609_GSM733610 | B |
| GSE29609_GSM733611 | B |
| GSE29609_GSM733612 | B |
| GSE29609_GSM733613 | B |
| GSE29609_GSM733614 | B |
| GSE29609_GSM733615 | A |
| GSE29609_GSM733616 | B |
| GSE29609_GSM733617 | B |

**Table S6**. The m6Ascore quantification results of the individuals in the merged cohort.

| **id** | **m6Ascore** |
| --- | --- |
| TCGA_TCGA-CJ-4920 | -2.549011675 |
| TCGA_TCGA-BP-5177 | 25.94150797 |
| TCGA_TCGA-B0-4842 | 68.32944331 |
| TCGA_TCGA-BP-4343 | -22.75636367 |
| TCGA_TCGA-MW-A4EC | -11.08122075 |
| TCGA_TCGA-B8-4143 | 11.71058927 |
| TCGA_TCGA-CJ-5675 | -17.25083318 |
| TCGA_TCGA-BP-4760 | -33.79144326 |
| TCGA_TCGA-B0-5113 | -37.52521202 |
| TCGA_TCGA-BP-4340 | -34.54767174 |
| TCGA_TCGA-B8-A7U6 | -6.778835278 |
| TCGA_TCGA-B8-5551 | -1.55083805 |
| TCGA_TCGA-B0-5400 | 44.4133062 |
| TCGA_TCGA-BP-4994 | -27.85052721 |
| TCGA_TCGA-A3-3349 | -23.40111104 |
| TCGA_TCGA-AK-3455 | -26.06174231 |
| TCGA_TCGA-CW-5585 | -34.28276993 |
| TCGA_TCGA-BP-4981 | 76.4032066 |
| TCGA_TCGA-BP-4337 | 42.09630797 |
| TCGA_TCGA-CJ-5686 | -28.52514848 |
| TCGA_TCGA-A3-A8OV | -5.712312183 |
| TCGA_TCGA-AK-3447 | 73.30031007 |
| TCGA_TCGA-B0-5702 | 108.6452538 |
| TCGA_TCGA-CJ-4918 | 7.434256422 |
| TCGA_TCGA-B0-4848 | -25.62855906 |
| TCGA_TCGA-BP-5194 | -19.67435579 |
| TCGA_TCGA-B2-5635 | -23.24282294 |
| TCGA_TCGA-B0-5096 | -16.67767849 |
| TCGA_TCGA-CZ-5986 | -53.53116431 |
| TCGA_TCGA-A3-3380 | -8.047275877 |
| TCGA_TCGA-BP-4329 | 4.573164553 |
| TCGA_TCGA-B0-4712 | 23.31814316 |
| TCGA_TCGA-B0-4691 | 43.72971043 |
| TCGA_TCGA-CZ-5982 | -43.60838866 |
| TCGA_TCGA-BP-4962 | 32.80627802 |
| TCGA_TCGA-CZ-5466 | 9.913261026 |
| TCGA_TCGA-AK-3450 | -25.58002277 |
| TCGA_TCGA-CJ-4876 | 1.322991039 |
| TCGA_TCGA-B0-4834 | 9.206973471 |
| TCGA_TCGA-B0-5713 | -28.85851982 |
| TCGA_TCGA-BP-5009 | 47.2801562 |
| TCGA_TCGA-CJ-4900 | 55.95246295 |
| TCGA_TCGA-BP-5186 | -29.10964743 |
| TCGA_TCGA-A3-3370 | -26.80140401 |
| TCGA_TCGA-B0-4810 | 0.947363737 |
| TCGA_TCGA-CJ-4916 | 24.62132866 |
| TCGA_TCGA-B0-4846 | -38.84108146 |
| TCGA_TCGA-CJ-4638 | 65.57516054 |
| TCGA_TCGA-BP-5184 | -13.99453757 |
| TCGA_TCGA-B0-5116 | 5.679654392 |
| TCGA_TCGA-CZ-4857 | -4.546601865 |
| TCGA_TCGA-B0-4718 | 44.4845961 |
| TCGA_TCGA-B0-5696 | -4.80468454 |
| TCGA_TCGA-BP-4326 | 11.09198098 |
| TCGA_TCGA-CZ-4856 | -8.578139383 |
| TCGA_TCGA-BP-4972 | -20.04726671 |
| TCGA_TCGA-BP-5169 | 120.2965717 |
| TCGA_TCGA-B2-5633 | -21.1051191 |
| TCGA_TCGA-B0-5693 | -33.30694482 |
| TCGA_TCGA-BP-4969 | 39.61409764 |
| TCGA_TCGA-BP-4165 | 40.8731216 |
| TCGA_TCGA-CJ-4870 | 9.591547993 |
| TCGA_TCGA-B8-5552 | -31.38335183 |
| TCGA_TCGA-BP-4164 | -37.19309146 |
| TCGA_TCGA-BP-4987 | -23.20691476 |
| TCGA_TCGA-BP-4769 | -25.84098485 |
| TCGA_TCGA-BP-4161 | -20.86885379 |
| TCGA_TCGA-BP-5010 | 85.25352502 |
| TCGA_TCGA-B2-3923 | -24.02062747 |
| TCGA_TCGA-AK-3445 | 6.355204748 |
| TCGA_TCGA-B2-4102 | -35.20332422 |
| TCGA_TCGA-B0-5083 | 29.82988368 |
| TCGA_TCGA-B0-4697 | 74.08450882 |
| TCGA_TCGA-B2-3924 | -11.99975349 |
| TCGA_TCGA-DV-A4VZ | 31.66222985 |
| TCGA_TCGA-B0-4821 | 61.7629234 |
| TCGA_TCGA-B0-4813 | 52.36864162 |
| TCGA_TCGA-CZ-5459 | 18.58013022 |
| TCGA_TCGA-B0-5108 | -0.919651554 |
| TCGA_TCGA-CZ-4862 | -43.78014353 |
| TCGA_TCGA-MM-A564 | 60.29738041 |
| TCGA_TCGA-CJ-4881 | 24.35692758 |
| TCGA_TCGA-B0-5077 | 5.02363304 |
| TCGA_TCGA-B0-5092 | 34.7634946 |
| TCGA_TCGA-EU-5905 | -23.91305222 |
| TCGA_TCGA-BP-5168 | -3.241176143 |
| TCGA_TCGA-B4-5832 | -64.8387845 |
| TCGA_TCGA-B8-5163 | -19.63269154 |
| TCGA_TCGA-DV-5575 | -23.18394488 |
| TCGA_TCGA-CW-5580 | -40.11524154 |
| TCGA_TCGA-B0-5692 | 6.350033912 |
| TCGA_TCGA-CJ-4640 | -19.95480656 |
| TCGA_TCGA-A3-3316 | 2.192939145 |
| TCGA_TCGA-BP-4797 | -9.074482975 |
| TCGA_TCGA-BP-5195 | -3.626021981 |
| TCGA_TCGA-B0-5691 | -41.39949407 |
| TCGA_TCGA-BP-5006 | -1.981037702 |
| TCGA_TCGA-CZ-5469 | 38.89052869 |
| TCGA_TCGA-BP-4999 | -17.57522486 |
| TCGA_TCGA-EU-5904 | -23.61607492 |
| TCGA_TCGA-BP-5189 | -21.40161109 |
| TCGA_TCGA-B0-5812 | -35.71296419 |
| TCGA_TCGA-A3-3385 | -22.60600871 |
| TCGA_TCGA-BP-4787 | 4.735906111 |
| TCGA_TCGA-BP-4325 | -23.17297389 |
| TCGA_TCGA-CJ-5677 | 24.86743354 |
| TCGA_TCGA-BP-4167 | 68.51963135 |
| TCGA_TCGA-CW-6097 | -32.9259857 |
| TCGA_TCGA-B8-5162 | 15.04485654 |
| TCGA_TCGA-CJ-6031 | 26.87655557 |
| TCGA_TCGA-CJ-4639 | -44.81573562 |
| TCGA_TCGA-BP-4759 | 58.94370578 |
| TCGA_TCGA-B8-4621 | 2.888903409 |
| TCGA_TCGA-CZ-5452 | 7.849412474 |
| TCGA_TCGA-B0-5699 | -31.20198894 |
| TCGA_TCGA-CZ-5460 | 22.36319739 |
| TCGA_TCGA-B0-4845 | 11.78044653 |
| TCGA_TCGA-MM-A563 | 18.05374881 |
| TCGA_TCGA-BP-4971 | 62.36732119 |
| TCGA_TCGA-B0-5712 | -10.55973656 |
| TCGA_TCGA-B0-4706 | 29.25106593 |
| TCGA_TCGA-CZ-5461 | -28.65297267 |
| TCGA_TCGA-6D-AA2E | 11.85547276 |
| TCGA_TCGA-EU-5907 | -45.89113881 |
| TCGA_TCGA-A3-3323 | -14.23882428 |
| TCGA_TCGA-CJ-4895 | -0.506382391 |
| TCGA_TCGA-CW-5591 | -47.39685781 |
| TCGA_TCGA-BP-4799 | 66.7041365 |
| TCGA_TCGA-B0-4847 | 75.22742128 |
| TCGA_TCGA-CW-6087 | -32.01273875 |
| TCGA_TCGA-BP-4173 | -9.622591823 |
| TCGA_TCGA-B8-4619 | -2.217398529 |
| TCGA_TCGA-BP-4176 | -17.23627832 |
| TCGA_TCGA-BP-4768 | -7.5439034 |
| TCGA_TCGA-B8-A54I | 76.25212843 |
| TCGA_TCGA-BP-5192 | -23.60576907 |
| TCGA_TCGA-BP-5181 | -20.81479116 |
| TCGA_TCGA-EU-5906 | -48.00331718 |
| TCGA_TCGA-B8-4620 | 8.900742407 |
| TCGA_TCGA-BP-4959 | -1.389885256 |
| TCGA_TCGA-CJ-4905 | -25.96939129 |
| TCGA_TCGA-CJ-6032 | -34.97978069 |
| TCGA_TCGA-B0-5703 | 24.64588902 |
| TCGA_TCGA-B8-A54F | -20.64391666 |
| TCGA_TCGA-B0-5095 | -12.54991046 |
| TCGA_TCGA-CZ-4861 | -12.62359569 |
| TCGA_TCGA-BP-4964 | -1.468215356 |
| TCGA_TCGA-B0-5402 | -17.72811742 |
| TCGA_TCGA-CJ-4891 | 67.32493454 |
| TCGA_TCGA-B0-4690 | -16.17144385 |
| TCGA_TCGA-A3-3374 | 61.56835383 |
| TCGA_TCGA-CZ-5470 | 6.577462848 |
| TCGA_TCGA-CZ-5463 | -52.40746344 |
| TCGA_TCGA-B8-4153 | 46.91009239 |
| TCGA_TCGA-B0-4698 | -12.97415953 |
| TCGA_TCGA-B0-5106 | 11.44754423 |
| TCGA_TCGA-CJ-4892 | -7.037401456 |
| TCGA_TCGA-CZ-4863 | -35.50317597 |
| TCGA_TCGA-BP-4330 | -19.378636 |
| TCGA_TCGA-B0-4824 | -7.278443285 |
| TCGA_TCGA-CJ-4871 | -9.978559831 |
| TCGA_TCGA-B0-5690 | -9.064889586 |
| TCGA_TCGA-B8-5549 | -20.73138466 |
| TCGA_TCGA-DV-A4W0 | -27.23161704 |
| TCGA_TCGA-CZ-5989 | -75.94948404 |
| TCGA_TCGA-B0-5081 | 48.10884738 |
| TCGA_TCGA-CZ-4866 | -38.50399976 |
| TCGA_TCGA-BP-5182 | -1.579654195 |
| TCGA_TCGA-CJ-6033 | 6.38078949 |
| TCGA_TCGA-BP-4327 | 23.94158402 |
| TCGA_TCGA-BP-5200 | -13.74756409 |
| TCGA_TCGA-BP-4160 | -26.62016663 |
| TCGA_TCGA-B0-5120 | -11.77546103 |
| TCGA_TCGA-B0-5100 | 36.42287606 |
| TCGA_TCGA-B0-4945 | -22.24308156 |
| TCGA_TCGA-BP-4781 | -32.69674426 |
| TCGA_TCGA-CJ-5684 | -4.484113123 |
| TCGA_TCGA-BP-4346 | -0.31016548 |
| TCGA_TCGA-AK-3426 | 87.73813394 |
| TCGA_TCGA-CJ-5682 | 3.880125582 |
| TCGA_TCGA-A3-A6NL | -17.0127684 |
| TCGA_TCGA-CZ-5987 | 17.8307679 |
| TCGA_TCGA-A3-3382 | -43.85772344 |
| TCGA_TCGA-CJ-4636 | 21.30055522 |
| TCGA_TCGA-CW-5583 | -50.76387838 |
| TCGA_TCGA-B0-4849 | -1.215321895 |
| TCGA_TCGA-B0-4852 | -16.8462736 |
| TCGA_TCGA-BP-4995 | -29.1918788 |
| TCGA_TCGA-BP-4798 | 18.87473841 |
| TCGA_TCGA-B0-5700 | 21.69833582 |
| TCGA_TCGA-AK-3456 | -13.16114594 |
| TCGA_TCGA-CJ-4641 | -19.65097856 |
| TCGA_TCGA-BP-4335 | 39.66909599 |
| TCGA_TCGA-B8-4146 | -93.12576615 |
| TCGA_TCGA-B0-4694 | 3.553165555 |
| TCGA_TCGA-B0-4836 | 2.551242727 |
| TCGA_TCGA-CJ-4635 | -16.07509627 |
| TCGA_TCGA-BP-4975 | -19.58374674 |
| TCGA_TCGA-B8-A54E | -7.376228841 |
| TCGA_TCGA-A3-3376 | -31.74145559 |
| TCGA_TCGA-BP-4758 | 61.25477285 |
| TCGA_TCGA-B0-5121 | 69.20889638 |
| TCGA_TCGA-BP-5180 | 17.58264799 |
| TCGA_TCGA-DV-5566 | -4.906886852 |
| TCGA_TCGA-BP-4784 | -19.89141114 |
| TCGA_TCGA-A3-3331 | -24.81352676 |
| TCGA_TCGA-CZ-5455 | -47.86903652 |
| TCGA_TCGA-A3-3383 | 26.5329987 |
| TCGA_TCGA-CJ-4875 | 62.70954444 |
| TCGA_TCGA-B0-4811 | 87.19400907 |
| TCGA_TCGA-BP-4341 | 33.01588503 |
| TCGA_TCGA-B0-5099 | -7.187046603 |
| TCGA_TCGA-B0-4839 | 63.76780396 |
| TCGA_TCGA-CJ-5678 | 18.45541466 |
| TCGA_TCGA-DV-A4VX | 31.24037831 |
| TCGA_TCGA-CJ-6030 | -12.59631179 |
| TCGA_TCGA-B0-4818 | -2.67471627 |
| TCGA_TCGA-B0-5706 | 25.38702196 |
| TCGA_TCGA-BP-4973 | -8.881503546 |
| TCGA_TCGA-B8-5165 | -33.72344621 |
| TCGA_TCGA-CJ-4901 | 37.4469684 |
| TCGA_TCGA-B0-5110 | -40.65972384 |
| TCGA_TCGA-B0-4838 | -15.38963611 |
| TCGA_TCGA-BP-4968 | 18.61141847 |
| TCGA_TCGA-A3-A8OX | -20.85850823 |
| TCGA_TCGA-A3-A6NN | -22.93178251 |
| TCGA_TCGA-B0-4814 | -11.6439633 |
| TCGA_TCGA-B0-4823 | -16.77739897 |
| TCGA_TCGA-B0-5098 | -9.118694197 |
| TCGA_TCGA-B0-5711 | -31.15692878 |
| TCGA_TCGA-A3-3322 | -4.341838934 |
| TCGA_TCGA-B8-4151 | -18.52273534 |
| TCGA_TCGA-BP-4982 | -33.80130045 |
| TCGA_TCGA-B0-4713 | 39.6582277 |
| TCGA_TCGA-CJ-4902 | 10.80525963 |
| TCGA_TCGA-B0-5399 | -9.047521189 |
| TCGA_TCGA-B0-4707 | 72.16747132 |
| TCGA_TCGA-CZ-4865 | -14.20916634 |
| TCGA_TCGA-BP-4344 | -16.67145577 |
| TCGA_TCGA-DV-5565 | 36.45207247 |
| TCGA_TCGA-AK-3436 | 15.04064045 |
| TCGA_TCGA-A3-A6NJ | -16.0689405 |
| TCGA_TCGA-BP-5191 | 117.8553043 |
| TCGA_TCGA-B8-4154 | -9.697803361 |
| TCGA_TCGA-B0-4688 | -22.17097341 |
| TCGA_TCGA-BP-4986 | -22.3543141 |
| TCGA_TCGA-B0-5075 | 1.734601624 |
| TCGA_TCGA-BP-4775 | -35.13548832 |
| TCGA_TCGA-CJ-4888 | 23.42773578 |
| TCGA_TCGA-AK-3431 | -20.82252329 |
| TCGA_TCGA-BP-4338 | 16.95852012 |
| TCGA_TCGA-B0-4701 | -9.847788628 |
| TCGA_TCGA-A3-3325 | -6.969564439 |
| TCGA_TCGA-CW-6090 | -11.26243055 |
| TCGA_TCGA-BP-4177 | -24.29211516 |
| TCGA_TCGA-BP-4170 | -13.37700155 |
| TCGA_TCGA-A3-3357 | -11.40714244 |
| TCGA_TCGA-BP-4960 | 82.16292915 |
| TCGA_TCGA-AK-3440 | 3.750538503 |
| TCGA_TCGA-A3-3365 | -31.39969711 |
| TCGA_TCGA-DV-5569 | -11.53623231 |
| TCGA_TCGA-BP-5170 | 18.77672543 |
| TCGA_TCGA-CJ-6027 | 7.756070374 |
| TCGA_TCGA-BP-4983 | -7.098980593 |
| TCGA_TCGA-B4-5378 | -39.67287649 |
| TCGA_TCGA-A3-3317 | -22.32460463 |
| TCGA_TCGA-BP-4771 | 32.09473905 |
| TCGA_TCGA-A3-3352 | -25.09084844 |
| TCGA_TCGA-B0-5084 | 38.51589246 |
| TCGA_TCGA-BP-4159 | 50.8948341 |
| TCGA_TCGA-B8-A54J | -2.812998955 |
| TCGA_TCGA-CJ-4644 | -29.10555342 |
| TCGA_TCGA-B4-5836 | -43.22126845 |
| TCGA_TCGA-B0-5117 | 74.28395005 |
| TCGA_TCGA-CJ-5680 | -26.13094733 |
| TCGA_TCGA-CW-5589 | -24.51408344 |
| TCGA_TCGA-BP-5000 | 67.89393525 |
| TCGA_TCGA-A3-3346 | 13.60414969 |
| TCGA_TCGA-CJ-5689 | 15.62096192 |
| TCGA_TCGA-3Z-A93Z | 0.806436458 |
| TCGA_TCGA-BP-4807 | -32.35505257 |
| TCGA_TCGA-CJ-4908 | -14.61331075 |
| TCGA_TCGA-BP-4352 | 48.59986055 |
| TCGA_TCGA-A3-A8OU | -1.772362473 |
| TCGA_TCGA-AK-3434 | 14.59403681 |
| TCGA_TCGA-CJ-5683 | 3.265649489 |
| TCGA_TCGA-A3-3326 | -4.395106356 |
| TCGA_TCGA-CJ-5671 | 20.51499438 |
| TCGA_TCGA-AK-3425 | 10.28352444 |
| TCGA_TCGA-CZ-4858 | -2.132939332 |
| TCGA_TCGA-B0-4841 | 127.0996757 |
| TCGA_TCGA-CJ-4897 | -21.1871092 |
| TCGA_TCGA-BP-4776 | 57.73896066 |
| TCGA_TCGA-B4-5377 | -47.74668321 |
| TCGA_TCGA-CZ-5468 | 17.88688188 |
| TCGA_TCGA-CJ-4904 | -28.54282941 |
| TCGA_TCGA-BP-4756 | -0.76387646 |
| TCGA_TCGA-B0-5088 | 45.39958167 |
| TCGA_TCGA-B0-4837 | 39.91140089 |
| TCGA_TCGA-BP-5174 | 27.69050934 |
| TCGA_TCGA-BP-4803 | -5.130971786 |
| TCGA_TCGA-B0-5097 | -8.378816694 |
| TCGA_TCGA-B0-5119 | -30.66266461 |
| TCGA_TCGA-BP-4345 | 26.62397807 |
| TCGA_TCGA-AK-3453 | 69.06497632 |
| TCGA_TCGA-BP-4342 | 2.736023778 |
| TCGA_TCGA-B0-4844 | 30.82477804 |
| TCGA_TCGA-BP-4804 | 25.19386178 |
| TCGA_TCGA-CW-6088 | -55.22047588 |
| TCGA_TCGA-B0-5701 | -15.72899907 |
| TCGA_TCGA-CJ-5681 | -52.87521472 |
| TCGA_TCGA-A3-3319 | 3.593889476 |
| TCGA_TCGA-BP-5007 | -6.219366883 |
| TCGA_TCGA-B8-A54D | 15.07934936 |
| TCGA_TCGA-B0-4833 | 37.70890552 |
| TCGA_TCGA-BP-4347 | -9.527489791 |
| TCGA_TCGA-CW-5590 | -5.959701023 |
| TCGA_TCGA-B0-4822 | 87.61291031 |
| TCGA_TCGA-B0-4703 | -13.96768406 |
| TCGA_TCGA-BP-4770 | 16.22858245 |
| TCGA_TCGA-CJ-4637 | -9.28649454 |
| TCGA_TCGA-CJ-4885 | -17.19365858 |
| TCGA_TCGA-B8-5546 | -34.87613418 |
| TCGA_TCGA-B8-5545 | -35.06852324 |
| TCGA_TCGA-CJ-5676 | 52.48213027 |
| TCGA_TCGA-B0-5707 | -3.218370415 |
| TCGA_TCGA-BP-5190 | 42.9841916 |
| TCGA_TCGA-B0-5709 | -31.86977787 |
| TCGA_TCGA-B0-5109 | 20.52958499 |
| TCGA_TCGA-B4-5843 | -41.33111912 |
| TCGA_TCGA-B2-4098 | 25.61649891 |
| TCGA_TCGA-CJ-4893 | -24.08316625 |
| TCGA_TCGA-BP-4985 | 15.9039571 |
| TCGA_TCGA-BP-5001 | 67.11997097 |
| TCGA_TCGA-A3-3313 | 3.594864227 |
| TCGA_TCGA-BP-4169 | -14.12895467 |
| TCGA_TCGA-B8-5553 | -41.72924504 |
| TCGA_TCGA-AK-3443 | -18.6005897 |
| TCGA_TCGA-B0-4696 | -19.35075127 |
| TCGA_TCGA-DV-5576 | -25.59456027 |
| TCGA_TCGA-BP-4963 | -4.061473252 |
| TCGA_TCGA-BP-4354 | -24.00873774 |
| TCGA_TCGA-BP-4789 | -28.85917095 |
| TCGA_TCGA-CZ-5462 | -38.41891877 |
| TCGA_TCGA-B0-5705 | -31.92110253 |
| TCGA_TCGA-B0-4710 | -20.33290484 |
| TCGA_TCGA-BP-5004 | -30.12412907 |
| TCGA_TCGA-A3-3387 | -29.62631329 |
| TCGA_TCGA-BP-5175 | 109.5616052 |
| TCGA_TCGA-BP-4331 | -27.06452257 |
| TCGA_TCGA-BP-4174 | -37.92350546 |
| TCGA_TCGA-CJ-6028 | -22.89423864 |
| TCGA_TCGA-A3-3367 | -18.15345915 |
| TCGA_TCGA-A3-3351 | -35.21408785 |
| TCGA_TCGA-CJ-4890 | 17.18146005 |
| TCGA_TCGA-CW-5581 | -33.35888182 |
| TCGA_TCGA-B0-4815 | 21.73919088 |
| TCGA_TCGA-AK-3454 | 81.69162984 |
| TCGA_TCGA-BP-4782 | -13.11466009 |
| TCGA_TCGA-CZ-5451 | -18.80591835 |
| TCGA_TCGA-CJ-4878 | -5.853905461 |
| TCGA_TCGA-CJ-5672 | 22.53133026 |
| TCGA_TCGA-BP-4765 | -27.49046936 |
| TCGA_TCGA-BP-4970 | -31.17100551 |
| TCGA_TCGA-B0-4819 | 61.60347869 |
| TCGA_TCGA-G6-A5PC | 91.09641031 |
| TCGA_TCGA-BP-4992 | 110.7315582 |
| TCGA_TCGA-CZ-4864 | -32.40182285 |
| TCGA_TCGA-BP-5198 | 11.15136695 |
| TCGA_TCGA-BP-4349 | 20.14530984 |
| TCGA_TCGA-BP-4965 | -42.10084247 |
| TCGA_TCGA-B4-5835 | -8.206664835 |
| TCGA_TCGA-B0-5094 | -14.54493931 |
| TCGA_TCGA-DV-5567 | -37.72185418 |
| TCGA_TCGA-BP-4334 | 90.96537584 |
| TCGA_TCGA-BP-5187 | -16.76960325 |
| TCGA_TCGA-CZ-5988 | -30.45380942 |
| TCGA_TCGA-CJ-4894 | -23.86425347 |
| TCGA_TCGA-BP-4763 | -34.35893693 |
| TCGA_TCGA-B0-4828 | 24.0713163 |
| TCGA_TCGA-CJ-5679 | 41.26374932 |
| TCGA_TCGA-CJ-4869 | 28.5282613 |
| TCGA_TCGA-A3-3363 | -29.67241367 |
| TCGA_TCGA-BP-4777 | 10.88674382 |
| TCGA_TCGA-B0-4843 | 72.97825351 |
| TCGA_TCGA-BP-4766 | -22.73824258 |
| TCGA_TCGA-B8-A54H | -22.39919255 |
| TCGA_TCGA-BP-4774 | 10.56405961 |
| TCGA_TCGA-A3-3373 | -24.18772451 |
| TCGA_TCGA-CZ-4860 | -30.34261053 |
| TCGA_TCGA-AK-3451 | 45.32133416 |
| TCGA_TCGA-B0-5104 | -10.04794438 |
| TCGA_TCGA-BP-4351 | 14.31960641 |
| TCGA_TCGA-B2-4099 | -10.44255331 |
| TCGA_TCGA-B0-5694 | 24.18542005 |
| TCGA_TCGA-CJ-4903 | -3.153795216 |
| TCGA_TCGA-CZ-5457 | -14.46461064 |
| TCGA_TCGA-BP-4158 | 5.574908144 |
| TCGA_TCGA-A3-3320 | -47.31773059 |
| TCGA_TCGA-DV-5568 | 26.81010616 |
| TCGA_TCGA-CZ-5985 | -40.59331875 |
| TCGA_TCGA-BP-4166 | 13.35833131 |
| TCGA_TCGA-AS-3777 | 50.9016748 |
| TCGA_TCGA-CZ-5456 | -10.83061343 |
| TCGA_TCGA-BP-5183 | -6.547234215 |
| TCGA_TCGA-CZ-4854 | 3.179393307 |
| TCGA_TCGA-A3-3306 | -33.28599988 |
| TCGA_TCGA-B8-5158 | -18.78487548 |
| TCGA_TCGA-B0-5695 | -18.95389719 |
| TCGA_TCGA-BP-5176 | 16.25883418 |
| TCGA_TCGA-CZ-4859 | -44.27144943 |
| TCGA_TCGA-CW-5587 | -42.03087288 |
| TCGA_TCGA-B4-5844 | -34.06624148 |
| TCGA_TCGA-BP-4801 | -15.78277267 |
| TCGA_TCGA-A3-3307 | -37.82569006 |
| TCGA_TCGA-B4-5834 | -34.65235621 |
| TCGA_TCGA-G6-A8L6 | 38.71528548 |
| TCGA_TCGA-BP-4991 | -30.99882155 |
| TCGA_TCGA-A3-3347 | -12.5331752 |
| TCGA_TCGA-B2-5636 | -46.16934917 |
| TCGA_TCGA-A3-3308 | -19.67774695 |
| TCGA_TCGA-MM-A84U | 56.42792439 |
| TCGA_TCGA-CJ-4907 | -18.79060121 |
| TCGA_TCGA-BP-5196 | -2.21780908 |
| TCGA_TCGA-CJ-4889 | -8.531274949 |
| TCGA_TCGA-AK-3428 | -35.05464775 |
| TCGA_TCGA-CJ-4874 | -23.37524508 |
| TCGA_TCGA-A3-3328 | -23.40594637 |
| TCGA_TCGA-CJ-4872 | 16.92179594 |
| TCGA_TCGA-B8-A54K | -21.06492191 |
| TCGA_TCGA-CJ-4873 | 71.25093499 |
| TCGA_TCGA-G6-A8L7 | 98.64802468 |
| TCGA_TCGA-CZ-5467 | -28.46079754 |
| TCGA_TCGA-B8-5159 | -46.04502266 |
| TCGA_TCGA-A3-3358 | -0.847933722 |
| TCGA_TCGA-A3-3372 | -32.25253189 |
| TCGA_TCGA-BP-4974 | -8.334347946 |
| TCGA_TCGA-CW-5584 | -25.11776872 |
| TCGA_TCGA-B4-5838 | -43.16233378 |
| TCGA_TCGA-B0-4699 | -27.16929021 |
| TCGA_TCGA-B8-5550 | -22.48802042 |
| TCGA_TCGA-A3-A8OW | -19.90990038 |
| TCGA_TCGA-BP-4967 | -5.457795241 |
| TCGA_TCGA-BP-5201 | 24.14569311 |
| TCGA_TCGA-T7-A92I | -45.07507497 |
| TCGA_TCGA-B0-5085 | 95.57257279 |
| TCGA_TCGA-CZ-4853 | -9.418153861 |
| TCGA_TCGA-A3-A6NI | -6.739299801 |
| TCGA_TCGA-AK-3465 | 24.30837588 |
| TCGA_TCGA-BP-4989 | 42.84867181 |
| TCGA_TCGA-G6-A8L8 | 14.80672026 |
| TCGA_TCGA-BP-4162 | -29.04943384 |
| TCGA_TCGA-BP-5173 | -6.590112313 |
| TCGA_TCGA-B8-A8YJ | -4.356502933 |
| TCGA_TCGA-B0-5080 | 2.006324349 |
| TCGA_TCGA-BP-4976 | -42.24455881 |
| TCGA_TCGA-BP-4163 | -22.98984256 |
| TCGA_TCGA-CJ-4899 | -30.77668803 |
| TCGA_TCGA-BP-4977 | -3.830527223 |
| TCGA_TCGA-A3-3335 | 12.34519197 |
| TCGA_TCGA-CJ-4634 | -35.61350846 |
| TCGA_TCGA-B2-4101 | -14.92912378 |
| TCGA_TCGA-BP-5185 | 134.8560683 |
| TCGA_TCGA-AK-3427 | 15.04152335 |
| TCGA_TCGA-BP-4998 | -24.41386843 |
| TCGA_TCGA-BP-5202 | -23.34761732 |
| TCGA_TCGA-B8-5164 | -47.0573837 |
| TCGA_TCGA-DV-5573 | -18.72731065 |
| TCGA_TCGA-B0-5697 | -11.4792604 |
| TCGA_TCGA-BP-4993 | 3.926686059 |
| TCGA_TCGA-B0-4817 | 58.61224839 |
| TCGA_TCGA-BP-4355 | 18.46011341 |
| TCGA_TCGA-A3-3359 | -31.80724618 |
| TCGA_TCGA-AK-3433 | 7.198524583 |
| TCGA_TCGA-B0-4827 | 4.034047636 |
| TCGA_TCGA-B2-A4SR | -31.042186 |
| TCGA_TCGA-B2-5641 | -19.38567317 |
| TCGA_TCGA-CJ-4882 | 45.00966101 |
| TCGA_TCGA-BP-4795 | -15.10511979 |
| TCGA_TCGA-CW-5588 | -16.35923434 |
| TCGA_TCGA-CZ-5464 | 7.644322952 |
| TCGA_TCGA-BP-4762 | -27.65220177 |
| TCGA_TCGA-BP-5178 | 68.85569356 |
| TCGA_TCGA-B0-5115 | -18.3674587 |
| TCGA_TCGA-B8-4622 | -9.761497393 |
| TCGA_TCGA-B0-5102 | 4.141020476 |
| TCGA_TCGA-BP-5008 | -16.6571852 |
| TCGA_TCGA-CJ-4642 | 59.58504498 |
| TCGA_TCGA-A3-3362 | -22.69597328 |
| TCGA_TCGA-B2-5639 | -15.70943431 |
| TCGA_TCGA-CZ-5458 | -16.94681549 |
| TCGA_TCGA-AS-3778 | -18.50571075 |
| TCGA_TCGA-B0-4693 | -24.57861911 |
| TCGA_TCGA-CJ-4884 | 12.64883107 |
| TCGA_TCGA-B0-5710 | -23.84049148 |
| TCGA_TCGA-BP-4353 | -31.42403898 |
| TCGA_TCGA-GK-A6C7 | -30.27355594 |
| TCGA_TCGA-BP-4761 | 71.38364718 |
| TCGA_TCGA-A3-3329 | -16.75856304 |
| TCGA_TCGA-BP-4332 | -14.94450121 |
| TCGA_TCGA-CJ-4643 | -28.93305263 |
| TCGA_TCGA-B8-A54G | -11.29877926 |
| TCGA_TCGA-BP-4961 | -31.31649474 |
| TCGA_TCGA-B0-5698 | -6.25016839 |
| TCGA_TCGA-BP-5199 | 14.43171512 |
| TCGA_TCGA-CW-6093 | -41.50527007 |
| TCGA_TCGA-B0-4700 | 37.34446881 |
| TCGA_TCGA-AK-3460 | -1.878278633 |
| TCGA_TCGA-AK-3458 | 5.420431696 |
| TCGA_TCGA-CZ-5984 | -19.75740182 |
| TCGA_TCGA-A3-3324 | -9.606090561 |
| TCGA_TCGA-CJ-4912 | 73.36218797 |
| TCGA_TCGA-CZ-5453 | 5.479515043 |
| TCGA_TCGA-A3-3378 | -14.86269666 |
| TCGA_TCGA-A3-3343 | -16.24084274 |
| TCGA_TCGA-CJ-4887 | 36.40735345 |
| TCGA_TCGA-DV-5574 | -2.614995506 |
| TCGA_TCGA-A3-A8CQ | -31.71759805 |
| TCGA_TCGA-B0-4816 | 3.46139851 |
| TCGA_TCGA-A3-3311 | -17.64702112 |
| TCGA_TCGA-CJ-4868 | 4.79888269 |
| TCGA_TCGA-B8-4148 | -30.71916851 |
| TCGA_TCGA-CJ-4886 | -26.76390252 |
| TCGA_TCGA-AK-3429 | -6.543511164 |
| TCGA_TCGA-CZ-5465 | -24.91229089 |
| TCGA_TCGA-B0-4714 | 41.56111879 |
| TCGA_TCGA-AK-3461 | -26.10260608 |
| TCGA_TCGA-CZ-5454 | -5.598379386 |
| TCGA_TCGA-BP-4790 | -29.75240551 |
| TCGA_TCGA-B0-5107 | 26.593841 |
| GSE29609_GSM733579 | 15.16827097 |
| GSE29609_GSM733580 | -20.25517082 |
| GSE29609_GSM733581 | -4.400126278 |
| GSE29609_GSM733582 | -13.9231558 |
| GSE29609_GSM733583 | 3.702148529 |
| GSE29609_GSM733584 | 6.2042015 |
| GSE29609_GSM733585 | 10.57644564 |
| GSE29609_GSM733586 | -8.297250151 |
| GSE29609_GSM733587 | 14.27989459 |
| GSE29609_GSM733588 | 9.629547243 |
| GSE29609_GSM733589 | -7.355152384 |
| GSE29609_GSM733590 | 5.707096741 |
| GSE29609_GSM733591 | 3.995357797 |
| GSE29609_GSM733592 | -9.868084942 |
| GSE29609_GSM733593 | 2.439529276 |
| GSE29609_GSM733594 | 8.926590145 |
| GSE29609_GSM733595 | -1.33838152 |
| GSE29609_GSM733596 | 5.086352225 |
| GSE29609_GSM733597 | -2.642494842 |
| GSE29609_GSM733598 | -13.69373374 |
| GSE29609_GSM733599 | 3.345049121 |
| GSE29609_GSM733600 | -1.995997633 |
| GSE29609_GSM733601 | 1.178007781 |
| GSE29609_GSM733602 | 12.17749204 |
| GSE29609_GSM733603 | -5.588119192 |
| GSE29609_GSM733604 | -1.335809968 |
| GSE29609_GSM733605 | -24.39517833 |
| GSE29609_GSM733606 | 9.372927528 |
| GSE29609_GSM733607 | -5.651706198 |
| GSE29609_GSM733608 | 2.656112001 |
| GSE29609_GSM733609 | 0.383735993 |
| GSE29609_GSM733610 | 3.500203721 |
| GSE29609_GSM733611 | 1.865616787 |
| GSE29609_GSM733612 | 1.386728481 |
| GSE29609_GSM733613 | 10.21127188 |
| GSE29609_GSM733614 | 2.340943558 |
| GSE29609_GSM733615 | -3.331597283 |
| GSE29609_GSM733616 | 4.370887925 |
| GSE29609_GSM733617 | -10.43902349 |
